# Design, synthesis, in vitro, and in silico evaluations of benzo[d]imidazole-amide-1,2,3-triazole-*N*-arylacetamide hybrids as new antidiabetic agents targeting α-glucosidase

**DOI:** 10.1038/s41598-023-39424-8

**Published:** 2023-07-31

**Authors:** Faeze Yousefnejad, Mahyar Mohammadi-Moghadam-Goozali, Mohammad Hosein Sayahi, Mohammad Halimi, Ali Moazzam, Maryam Mohammadi-Khanaposhtani, Somayeh Mojtabavi, Mehdi Asadi, Mohammad Ali Faramarzi, Bagher Larijani, Massoud Amanlou, Mohammad Mahdavi

**Affiliations:** 1 Endocrinology and Metabolism Research Center, Endocrinology and Metabolism Clinical Sciences Institute, Tehran University of Medical Sciences, Tehran, Iran; 2 Department of Medicinal Chemistry, Faculty of Pharmacy, Tehran University of Medical Sciences, Tehran, Iran; 3 Department of Chemistry, Payame Noor University, P.O. Box 19395-3697, Tehran, Iran; 4 Department of Biology, Islamic Azad University, Babol Branch, Babol, Iran; 5 Cellular and Molecular Biology Research Center, Health Research Institute, Babol University of Medical Sciences, Babol, Iran; 6 Department of Pharmaceutical Biotechnology, Faculty of Pharmacy, Tehran University of Medical Sciences, Tehran, Iran; 7 Department of Medicinal Chemistry, Faculty of Pharmacy, Iran University of Medical Sciences, Tehran, Iran

**Keywords:** Drug discovery, Medicinal chemistry

## Abstract

α-Glucosidase as a carbohydrate-hydrolase enzyme is a crucial therapeutic target for type 2 diabetes. In this work, benzo[d]imidazole-amide containing 1,2,3-triazole-*N*-arylacetamide derivatives **8a–n** were synthesized and evaluated for their inhibitory activity against α-glucosidase. In vitro α-glucosidase inhibition assay demonstrated that more than half of the title compounds with IC_50_ values in the range of 49.0–668.5 μM were more potent than standard inhibitor acarbose (IC_50_ = 750.0 µM). The most promising inhibitor was *N*-2-methylphenylacetamid derivative **8c**. Kinetic study revealed that compound **8c** (K_i_ = 40.0 µM) is a competitive inhibitor against α-glucosidase. Significantly, molecular docking and molecular dynamics studies on the most potent compound showed that this compound with a proper binding energy interacted with important amino acids of the α-glucosidase active site. Study on cytotoxicity of the most potent compounds **8c**, **8e**, and **8g** demonstrated that these compounds did not show cytotoxic activity against the cancer and normal cell lines MCF-7 and HDF, respectively. Furthermore, the ADMET study predicted that compound **8c** is likely to be orally active and non-cytotoxic.

## Introduction

Diabetes mellitus is a widespread disease that related to the carbohydrate metabolism defect. In this disease, the processes related to the production and effectiveness of insulin as the most important hormone for regulation of the metabolism of carbohydrates are disturbed. The insufficient insulin secretion in the type 1 diabetes mellitus and insulin resistance in the type 2 diabetes mellitus led to hyperglycemia^1^. Type 2 diabetes with prevalence around 90–95% is the most common form of diabetes mellitus^2^. In this type of diabetes, the most of the strategies for treatment are related to reducing the entry of glucose into the blood and increasing the excretion of glucose from the kidney^3^. In this regards, inhibition of carbohydrate hydrolase enzymes by suppressing the conversion of carbohydrates into glucose, controls the postprandial hyperglycemia^4^. One of the most important small intestinal enzymes that break down carbohydrates to glucose is α-glucosidase^5^. Inhibition of the latter enzyme is an important target for discovery of anti-diabetic agents^6^. Three drugs with the α-glucosidase inhibition mechanism are available in the pharmaceutical market for the treatment of diabetes: acarbose, miglitol, and voglibose^7^. The complexity of the synthesis of these compounds and their gastrointestinal side effects have encouraged medicinal chemists to search for new structures with α-glucosidase inhibition mechanism^8^.

**Figure 1:**
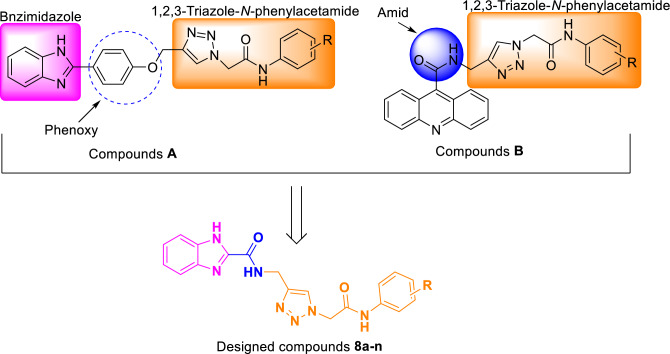
Design strategy for the new benzo[d]imidazole-amide-1,2,3-triazole-*N*-arylacetamide hybrids **8a–n**.

*N*-Heterocycles are an important class of heterocycles that are widely used in the design of new bioactive compounds^9–13^. Benzimidazole has a *N*-heterocycle and found in the various bioactive compounds with anticancer, antimicrobial, antiviral, and anti-diabetic properties^14^. Benzimidazole has attracted much attention for design of new structures with α-glucosidase inhibitory activity^15–17^. Recently, several series of synthetic benzimidazole-based α-glucosidase inhibitors such as compounds **A** have been introduced (Fig. 1)^18^. On the other hand, based on the recent findings, 1,2,3-triazole-*N*-arylacetamid moiety in connection with various heterocycles could effectively inhibit α-glucosidase^19–21^. In the compounds **A**, 1,2,3-triazole-*N*-arylacetamide moiety by a phenoxy group attached to benzimidazole ring and in the potent α-glucosidase inhibitors **B**, the latter moiety by an amid unit attached to acridine ring (Fig. 1)^22^. Therefore, it seems that connection of benzimidazole ring to 1,2,3-triazole-*N*-phenylacetamid moiety by a amid unit can led to α-glucosidase inhibitory potency. Therefore, a novel series of benzo[d]imidazole-amide-1,2,3-triazole-*N*-arylacetamide hybrids **8a–n** were designed, synthesized, and screened as potent α-glucosidase inhibitors. Kinetic analysis as well as in silico evaluations were performed on these new synthesized compounds.

## Results and discussion

### Chemistry

**Scheme 1.**
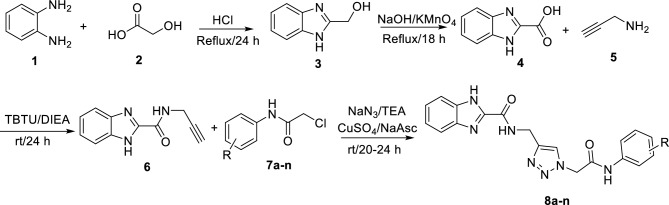
Synthetic procedure benzo[d]imidazole-amide-1,2,3-triazole-*N*-arylacetamide hybrids **8a–n**.

The synthesis of benzo[d]imidazole-amide-1,2,3-triazole-*N*-arylacetamide hybrids **8a–n** has been schematically shown in Scheme 1. Initially, a mixture of *o*-phenylenediamine **1** and glycolic acid **2** (60 mmol) in HCl was stirred at reflux condition for 24 h and 1*H*-benzo[d]imidazol-2-yl)methanol **3** was obtained. The latter compound in the presence of NaOH and KMno_4_ at reflux condition was converted to 1*H*-benzo[d]imidazole-2-carboxylic acid** 4**. Propargylted form of 1*H*-benzo[d]imidazole-2-carboxylic acid** 4**, compound **6**, was obtained of reaction between this acid and propargyl amine** 5** in the presence of TBTU and DIEA. Compound **6** was involved in a click reaction with chloride derivatives **7a–n** to give target benzo[d]imidazole-amide-1,2,3-triazole-*N*-arylacetamide hybrids **8a–n**^13^. All the mentioned steps are showed in Scheme 1. The obtained new structures **8a–n** were confirmed by spectroscopic techniques such as FTIR, ^1^H, and ^13^C NMR spectroscopy.

For example, in the ^1^H NMR spectrum of compound **8a**, hydrogen of imidazole appears in 13.30 ppm, hydrogen of NH amide appears in 10.44 ppm, hydrogen of NH amide (Propargyl amine) appears in 9.44 ppm, hydrogen of 1,2,3-triazole ring appears in 8.03 ppm, hydrogens of aromatic region appear between 7.73 and 7.08 ppm, hydrogens of CH_2_ group appear in 5.31 ppm, and hydrogens of CH_2_ group (Propargyl amine) appear in 4.61 ppm. In the ^13^C NMR spectrum of compound **8a**, carbons of amide groups appear in 64.71 and 159.27 ppm, carbons of C–N bonds appear in 145.95, 145.04, 142.98, 138.89, 134.96 ppm, carbons of aromatic region appear between 129.37 and 113.03 ppm, and carbons of CH_2_ group appear 52.64 and 35.03 ppm.

### Inhibitory activity of the new derivatives 8a–n against α-glucosidase

The newly synthesized benzo[d]imidazole-amide-1,2,3-triazole-*N*-arylacetamide hybrids **8a–n** were evaluated against yeast form of α-glucosidase. The obtained results presented in Table 1. These results revealed that more than half of the evaluated compounds with IC_50_ values of 49.0–668.5 μM inhibited the target enzyme better than the standard drug acarbose with IC_50_ value of 750.0 μM. As can be seen Table 1, derivation of designed scaffold was performed based on the change of substituents on phenyl ring of *N*-phenylacetamide moiety. The most active compound was 3-methyl derivative **8c** with IC_50_ value of 49.0 ± 0.4 µM. This compound was 15.3-fold more potent than positive control. Furthermore, compounds **8e**, **8g**, **8k**, and **8m** with 2,3-dimethyl, 2,6-dimethyl, 4-chloro and 4-fluoro substituents, respectively, exhibited a significant anti-α-glucosidase activity (IC_50_ values ≤ 183.6 ± 0.8 µM).

**Table 1:** In vitro α-glucosidase inhibitory activities of benzo[d]imidazole-amide-1,2,3-triazole-*N*-arylacetamide hybrids **8a–n**.


Compound	R	IC_50_ (µM)^a^
**8a**	H	> 750
**8b**	2-Methyl	> 750
**8c**	3-Methyl	49.0 ± 0.4
**8d**	4-Methyl	> 750
**8e**	2,3-Dimethyl	119.2 ± 0.8
**8f.**	2,4-Dimethyl	422.6 ± 1.1
**8g**	2,6-Dimethyl	125.6 ± 0.3
**8h**	4-Ethyl	251.9 ± 1.6
**8i**	4-Methoxy	> 750
**8j**	4-F	> 750
**8k**	4-Cl	134.2 ± 0.9
**8l**	2,4-Dichloro	668.5 ± 1.4
**8m**	4-Br	183.6 ± 0.8
**8n**	4-Nitro	> 750
Acarbose	–	750.0 ± 2.0

^a^Data are expressed as mean ± S.E. of at least three different experiments.

### Structure–activity relationships (SAR)

As can be seen in Table 1, based on SAR study, anti-α-glucosidase activity of compounds **8a–n** dramatically depended on type and position of substituents on pendant phenyl group. Obtained data demonstrated that un-substituted derivative **8a**, 2-methyl derivative **8b**, and 4-methyl derivative **8d** have not activity against target enzyme while 3-methyl derivative **8c** was the most potent compound among the all newly synthesized compounds. The replacement of 4-methyl substitution of compound **8b** with methoxy (compound **8i**), fluoro (compound **8j**), and nitro (compound **8n**) substitutions does not improve the inhibitory activity, but the replacement of 4-methyl with ethyl (compound **8h**), chloro (compound **8k**), and bromo (compound **8m**) substitutions improved the inhibitory activity (the order of activity: Cl > Br > Ethyl). Addition of the second chlorine substituent on the 2-position of the 4-chloro derivative **8k**, in case of compound **8l**, led to a significant decrease in the inhibitory activity. In the case of 2-methyl derivative **8b**, introduction of the second methyl group in any position, especially in positions 3 (compound **8e**) and 6 (compound **8g**), improved inhibitory activity.

### Comparison of the new compounds 8 with template compounds A and B

**Scheme 2.**
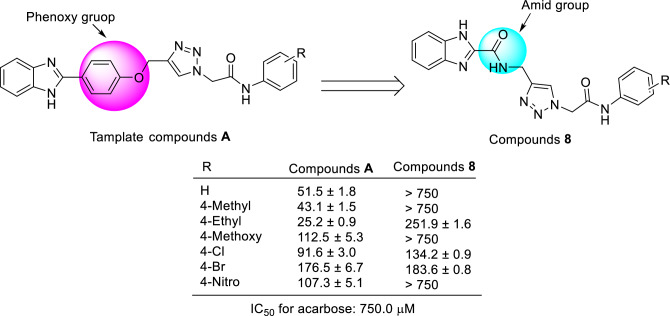
Comparison of IC_50_ values of new derivatives **8** against α-glucosidase with their corresponding analogs of template derivatives **A**^18^.

The comparison of IC_50_ values of the new derivatives **8** with their corresponding analogs of the template compounds **A** revealed that reported analogs **A** were more potent than their corresponding analogs of the new series **8** (Scheme 2)^18^. It should be noted that 4-chloro and 4-bromo derivatives of the new series **8** had inhibitory activity approximately similar to their corresponding analogs of the reported group **A**.

**Scheme 3.**
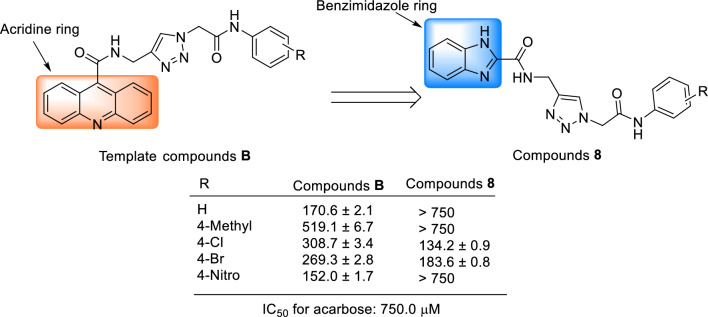
Comparison of the anti-α-glucosidase effects of new derivatives **8** with their corresponding analogs of template derivatives **B**^22^.

The comparison of the anti-α-glucosidase activity of the new derivatives **8** with their corresponding analogs of template compounds **B** revealed that 4-chloro and 4-bromo derivatives of new series **8** were more potent than their corresponding analogs of the reported series** B** (Scheme 3)^22^. In contrast, un-substituted, 4-methyl, and 4-nitro derivatives of the series** B** were more potent than their analogs of the series **8**. It should be noted that the most potent compound of the series **8** (3-methyl derivative) was more potent than the most potent compound of the series **B** (3-bromo derivative).

### Kinetic study

**Figure 2:**
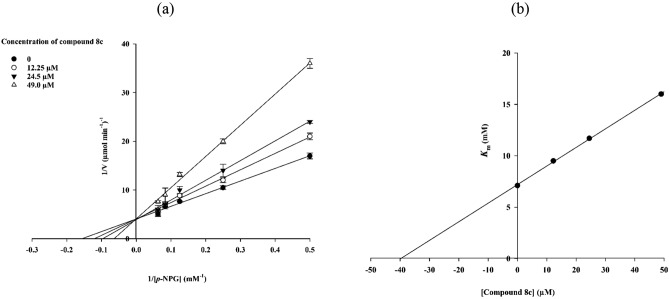
Inhibitory kinetics of compound **8c** on α-glucosidase. (**a**) Lineweaver–Burk plots for inhibition of compound **8c**. (**b**) The secondary plot of Lineweaver–Burk plots for determination K_i_ value of compound **8c**.

To evaluate of the inhibition mechanism, kinetic study was performed on the most active compound **8c**. As shown in Fig. 2a, the lines of Lineweaver–Burk plot with enhancement in the concentration of inhibitor **8c** had a fixed intercept on the Y-intercept and X-slopes. Therefore, values of V_max_ remained constant while the values of K_m_ increased. The obtained data showed that compound **8c** was a competitive inhibitor for α-glucosidase (Fig. 2a). Besides, the K_i_ value was 40.0 µM that was obtained by the secondary plot of Lineweaver–Burk plots (Fig. 2b).

### Docking study

**Figure 3:**
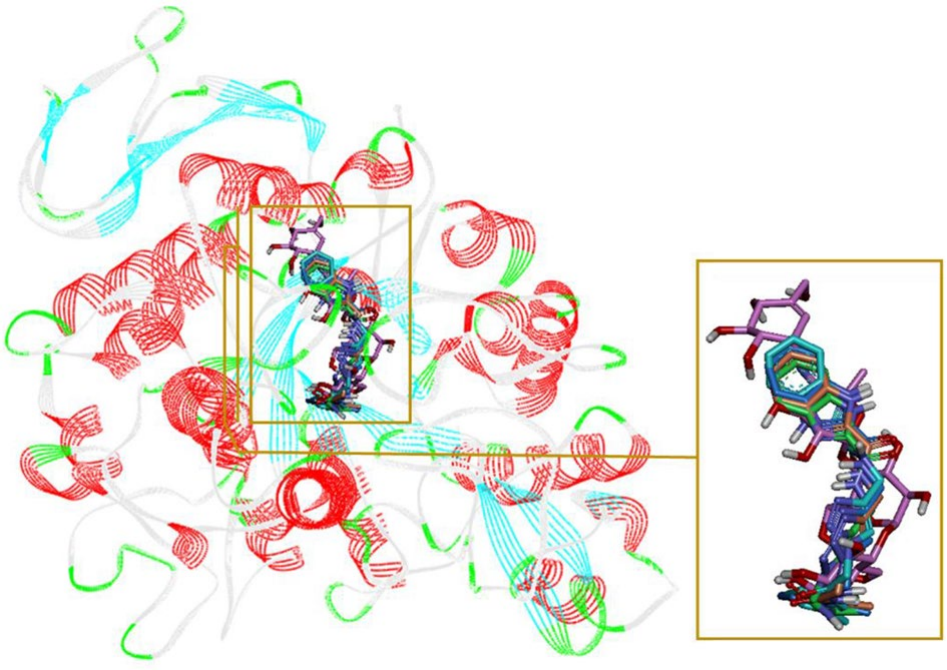
Acarbose (pink) and the selected compounds **8b** (orange), **8c** (cyan), and **8d** (green) superimposed in the α-glucosidase active site.

In order to explain interactions and to justify observed SAR, we selected three methyl derivatives **8b**, **8c**, and **8e** as representatives of the new synthesized compounds and performed a molecular docking study of them in the α-glucosidase active site^23^. The superposed structure of positive control acarbose and the selected compounds in the active site of α-glucosidase is shown in Fig. 3.

**Figure 4:**
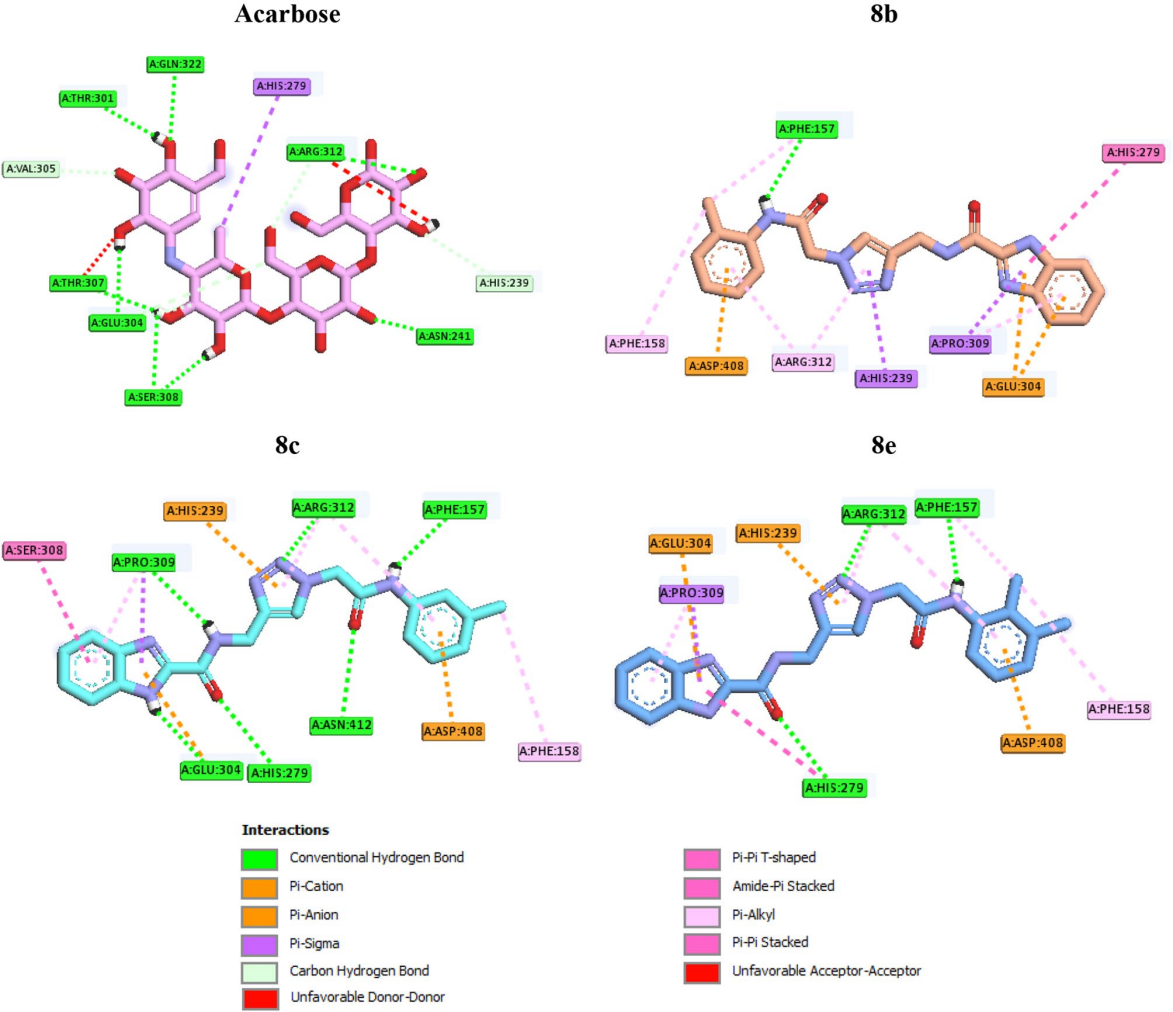
2D interaction modes of acarbose (**a**) and the selected compounds **8b** (**b**), **8c** (**c**), and **8e** (**d**) in the α-glucosidase active site.

Interaction modes of acarbose and compounds **8b**, **8c**, and **8e** are showed in the Fig. 4. As can be seen in this figure, acarbose created eight hydrogen bonds with active site residues Thr307, Asn241, Glu304, Ser308, Thr301, Pro309, Arg312, and Gln322. This standard inhibitor also formed a hydrophobic interaction with His279, non-classical hydrogen bonds with Val305 and His239 (two interactions), and unfavorable interactions with Thr307 and Arg312 (two interactions).

The most potent compound **8c**, with 3-methyl substituent on phenyl ring of *N*-phenylacetamid moiety, established six hydrogen bonds whit residues Pro309, Glu304, Asn412, His279, Arg312, and Phe157 (F. This compound created two π-anion interactions with Asp408 and Glu304 and a π-cation interaction with His239. Furthermore, several hydrophobic interactions between this compound and residues Ser308, Pro309, Arg312, and Phe158 were also observed. Addition of a methyl group at 2-position of 3-methyl derivative **8c**, as in case of 2,3-dimethyl derivatives **8e**, the inhibitory activity diminished to around 2.5 fold. A survey on the interaction modes of compounds **8c** and **8e** revealed that the mentioned addition led to elimination of three hydrogen bonds (Pro309, Glu304, and Asn412) in the interaction mode of the second potent compound **8e** in comparison to the most potent compound **8c**. Three π-ion interactions are in the both mentioned compounds same. Also, the number of hydrophobic interactions is the same in compounds **8c** and **8e**, only the type and number of amino acids participating in the interactions are slightly different. As can be seen in Fig. 4, compound **8c** formed six hydrophobic interactions with Ser308, Pro309, Arg312, and Phe158 while compound **8e** formed six hydrophobic interactions with His279, Pro309, Arg312, Phe157, and Phe158.

On the other hand, as can be seen in Table 1, changing the position of methyl group of 3-positon in compound **8c** to 2-posion, as in case of compound **8b**, abolished the inhibitory activity. Docking data demonstrated that inactive compound **8b** only formed a hydrogen bond with the active site residue Phe157. This compound also created π-anion interactions with residues Asp408 and Glu304 and hydrophobic interactions whit Phe158, Phe157, Arg312, His239, Pro309, and His279.

### Molecular dynamics

A ligand binding to a receptor is a dynamic event, like many other molecular interactions. Hence, simulating and then analyzing receptor-ligand motion in an environment containing water and ions, comparable to that of a natural environment, can be beneficial for understanding the complex stability and flexibility. According to in vitro studies, compound **8c** was the most potent inhibitor against α-glucosidase. Therefore, the docking files of this compound and acarbose as a standard inhibitor of α-glucosidase were simulated in an explicit hydration environment by molecular dynamics (MD) simulation and the result was interpreted for evaluating the stability and flexibility of the protein–ligand complex^24^. Simulation was performed in two steps. At first step simulation was performed for 10 ns and it was observed that both **8c** and acarbose were stable at the binding site of α-glucosidase. Therefore the simulation time was extended for 100 ns for better evaluation of the complex. Multiple tools were used to further analyze the simulation trajectory of these compounds.

**Figure 5:**
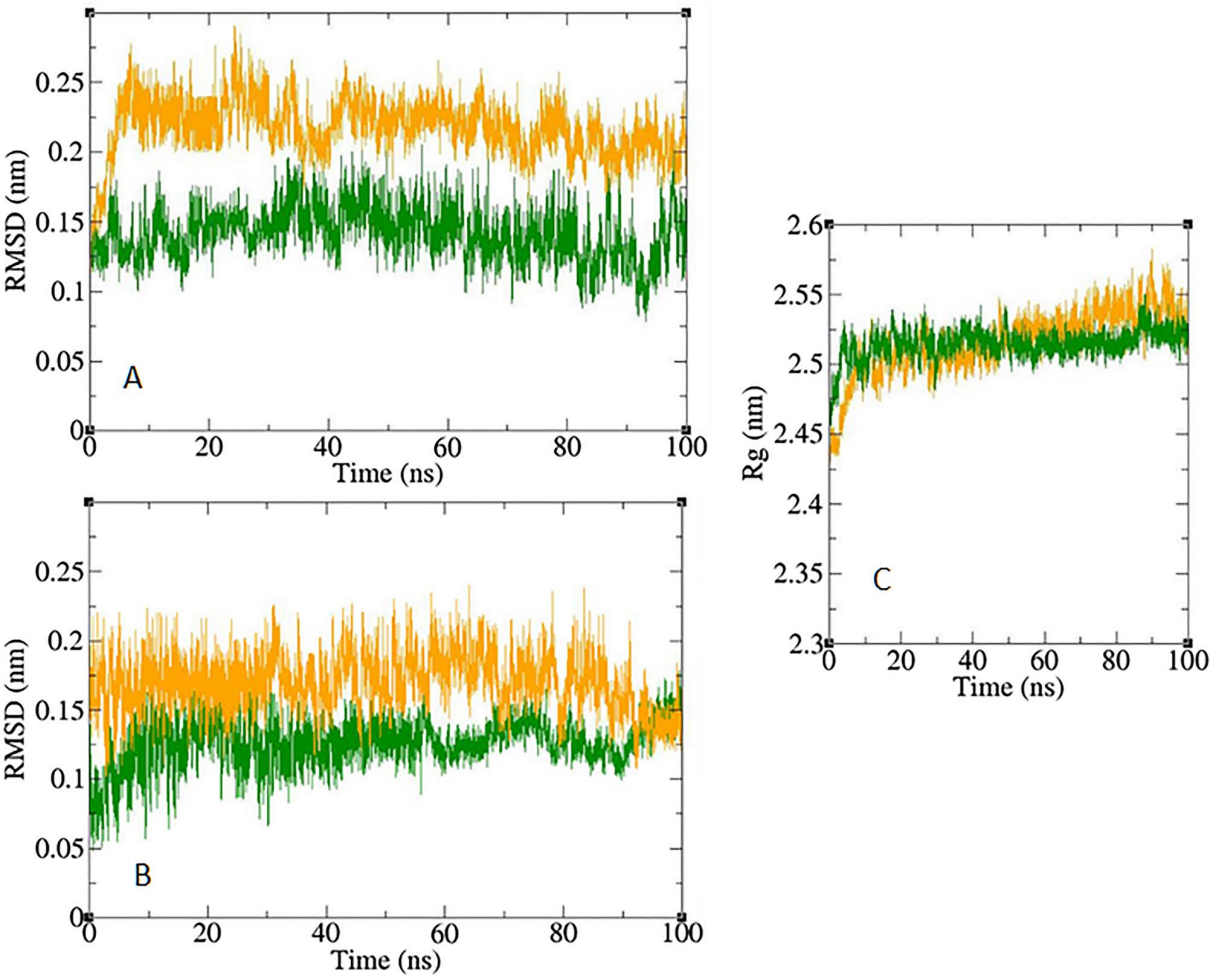
Superimposed RMSD of Cα atoms of α-glucosidase in complex with **8c** (orange) and acarbose (green) (**A**). Superimposed RMSD of **8c** (orange) and acarbose (green) in complex with α-glucosidase (**B**). Time dependence of the radius of gyration (Rg) graph of α-glucosidase in complex with **8c** (orange) and acarbose (green) (**C**).

**Figure 6:**
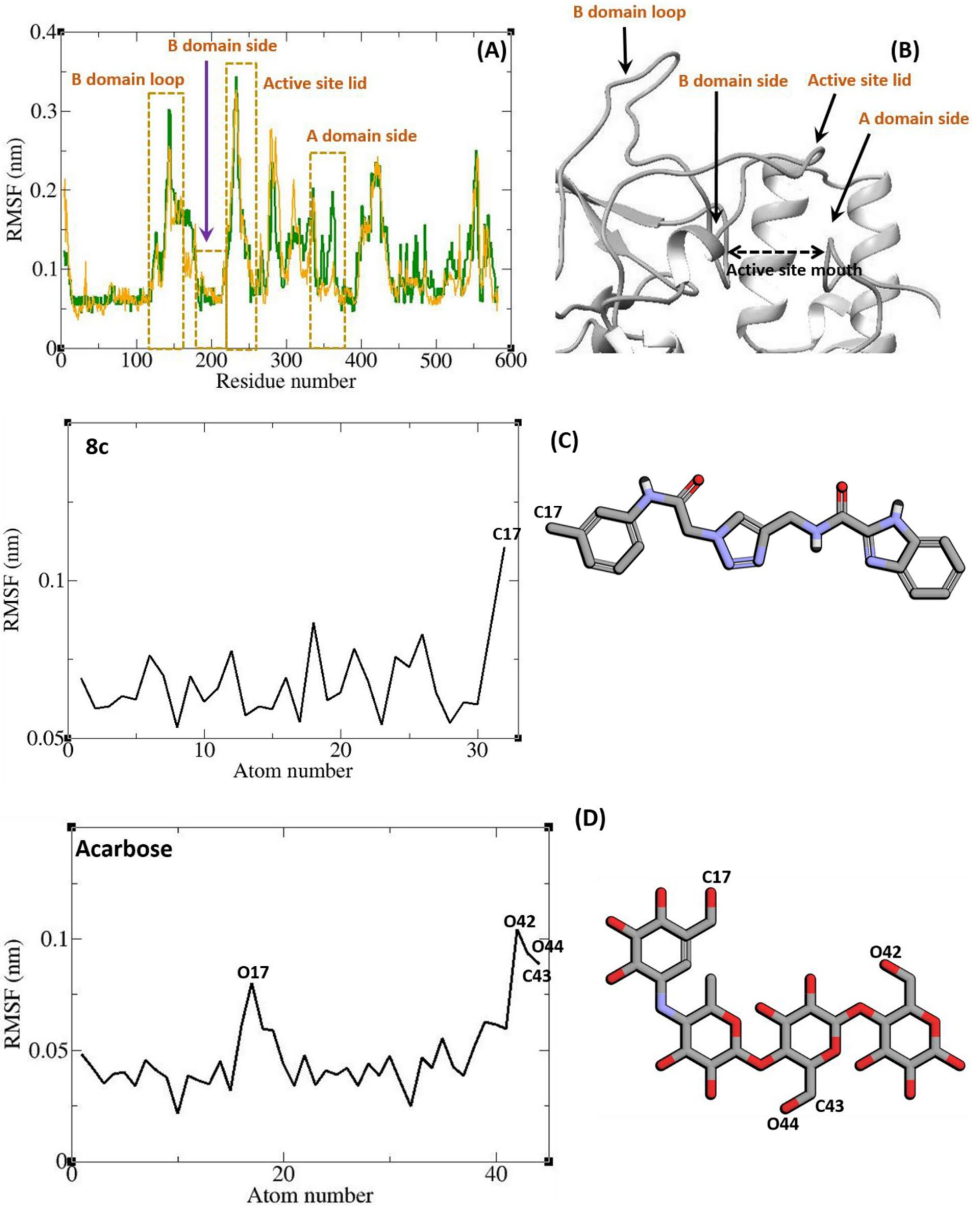
RMSF graph of the Cα atoms of α-glucosidase in complex with acarbose (green) and **8c** (orange) (**A**). Close-up representation of α-glucosidase active site (**B**). RMSF graph of the heavy atoms of **8c** (**C**) and acarbose (**D**) in complex with α-glucosidase. Structure of these compounds and parts of these molecules with greatest fluctuations are illustrated.

The result of the simulation was visualized by “Visual Molecular Dynamic” (VMD) that showed stable complexes during all the simulation time. For more evaluation of the stability of the complexes, root-mean-square d eviations (RMSDs) and radius of gyrations (Rgs) were calculated for all the saved structures during MD simulation, and changes in these factors during simulation were measured. For assessing residual flexibility during simulation, the root mean square fluctuation (RMSF) of the backbone atoms was also calculated. The results of these calculations are illustrated in Figs. 5 and 6. According to Fig. 5, the RMSD of α-glucosidase is less than 3 Å throughout the simulation which could be an indicator for a stable structure. The average RMSD value of α-glucosidase in the complex with acarbose and/or **8c** was 0.141 and 0.217 Å respectively. The RMSD of acarbose and **8c** in the complex with α-glucosidase were less than 3 Å too with an average RMSD of 0.120 and 0.167 Å, respectively. These results are indicator of the stability of the **8c** and acarbose in the active site of α-glucosidase during the simulation. Radius of gyration (Rg) is used for evaluation of compactness of protein during the simulation. The Rg value of α-glucosidase in all complexes was between a narrow range of 2.43–2.57 nm and did not show unusual change during the simulation time. The average Rg of α-glucosidase was 2.51 and 2.52 nm in the complex of α-glucosidase with acarbose and **8c**, respectively.

RMSF values of the Cα atoms of α-glucosidase is depicted in Fig. 6. According to this figure fluctuation of α-glucosidase atoms in complex with **8c** and acarbose are so similar. α-Glucosidase has several structural and functional domains and based on Fig. 6 the fluctuation of these parts are different. “A domain side” and “B domain side” show little fluctuations. It can be the result of the non-bond interactions of these domains with ligands. Residues located in loop regions i.e. “B domain loop” and “active site lid” show higher fluctuations as is expected for loops. The fluctuation of ligand atoms are depicted in Fig. 6. All atoms of **8c** and acarbose showed RMSF less than 2 Å. Ring atoms had very low fluctuation. Ring limits atomic fluctuation and at the same time several non-bond interactions like π–anion and π–π T-shaped, π–sigma, and hydrogen bond between the rings and binding site residues could be made that limit the fluctuation.

**Figure 7:**
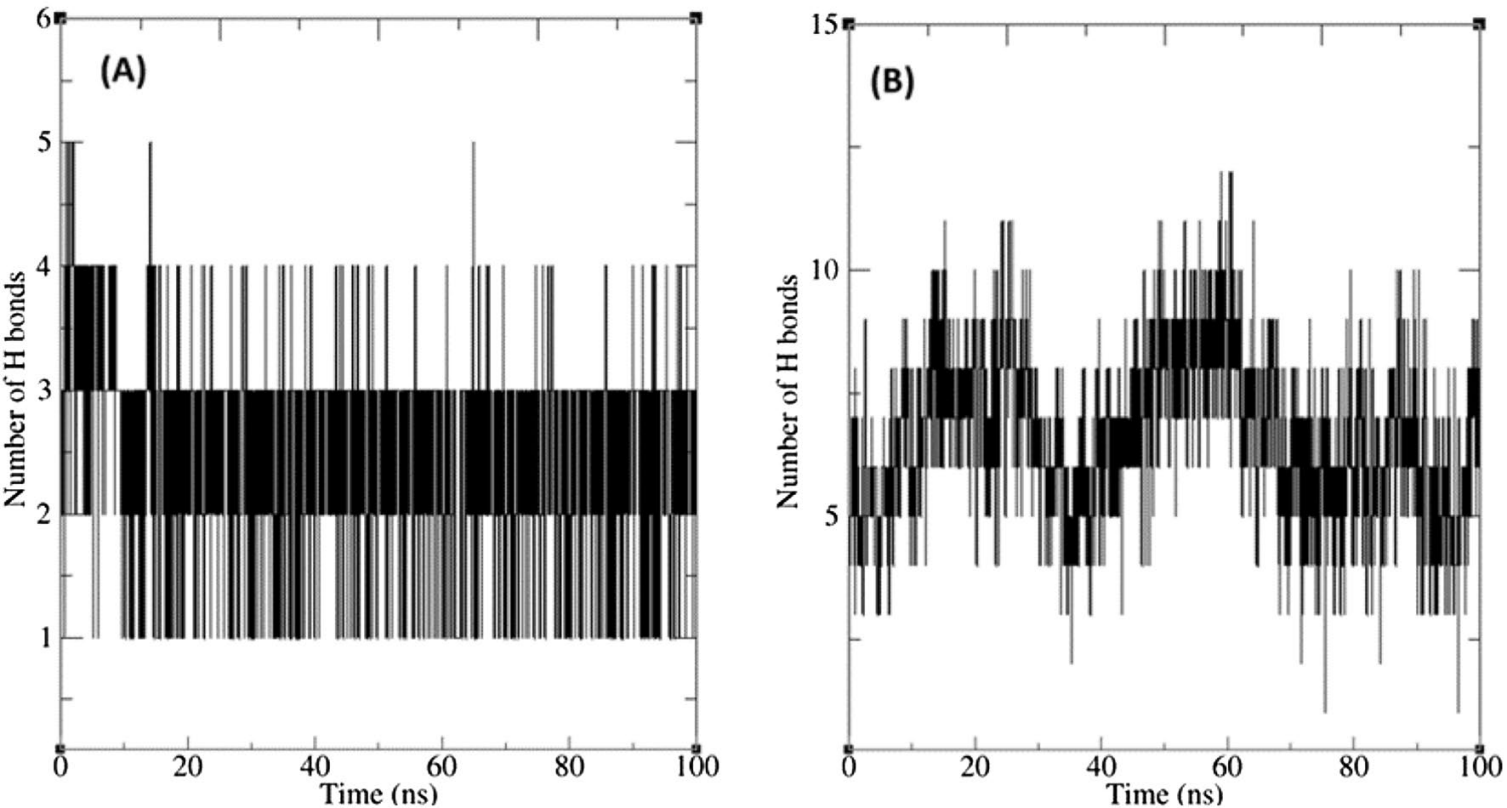
The numbers of hydrogen bonds between compound **8c** (**A**), and acarbose (**B**) with α-glucosidase binding site residues during MD simulation.

Analyzing the MD trajectories showed that the number of hydrogen bonds between ligands and α-glucosidase was constantly changing (Fig. 7). Accordingly, the number of hydrogen bonds in α-glucosidase–acarbose complex was changing mainly between 5 and 10 that could be an indicator of a strong complex. According to docking studies, compound **8c** makes 6 hydrogen bonds in the binding site of α-glucosidase (Fig. 4). However MD simulation showed that the number of hydrogen bonds in α-glucosidase-**8c** complex was mainly changing between 2 and 3. These differences between docking and MD simulation studies are not unexpected as the conformation of both ligand and the receptor fluctuates during the MD simulation, so a wide variety of interactions arise^25^. However, binding energy analysis in the next step demonstrated that the overall impact of these interactions was in favor of binding of **8c** to α-glucosidase.

### Binding free energy analysis

**Figure 8:**
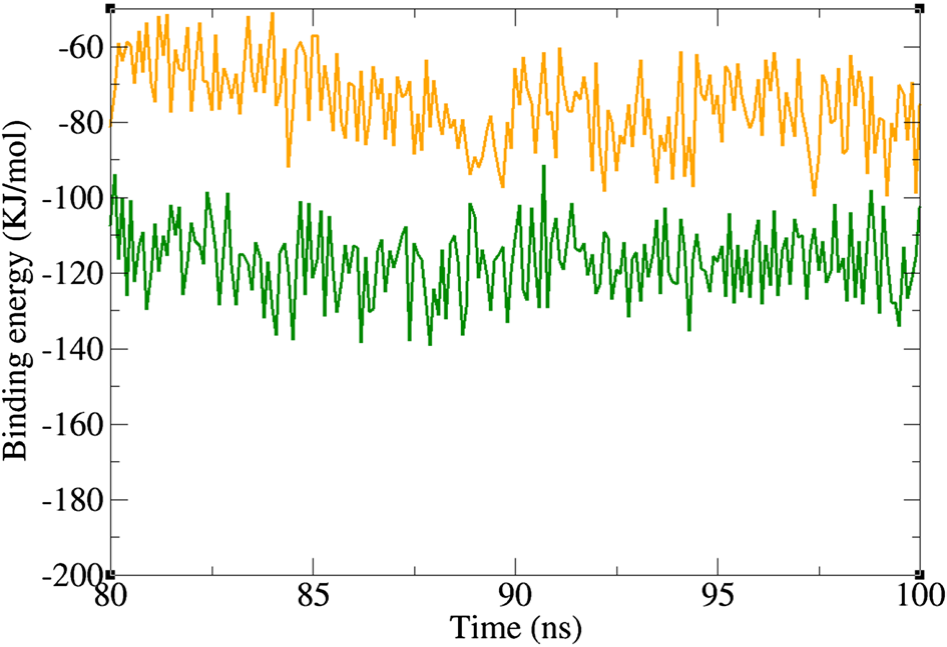
Diagram of binding energy changes during the last 20 ns of simulation time. α-glucosidase in complex with acarbose (green), and **8c** (orange).

Binding energy of a ligand to a protein receptor can be estimated by the molecular mechanic/Poisson–Boltzmann surface area (MM/PBSA) method. The nature of the dominant interactions in a ligand–receptor complex can be revealed by this method. The estimation of binding energy by molecular docking is not so accurate as there is only a single snapshot of a structure. However, in MD simulation, several snapshots of the complex could be provided in a period of time that leads to a more accurate estimation of binding energy. The result of free binding energy analysis is presented in Table 2. In this study, both acarbose and **8c** revealed negative binding energies. The average MM/PBSA free binding energy of the known inhibitor (acarbose) with α-glucosidase was − 115.7 kJ/mol, while **8c** exhibited − 75.1 kJ/mol binding free energy. Figure 8 shows the diagram of binding energy changes during the last 20 ns of MD simulation. In both complexes, binding energy fluctuates in a narrow negative range that is an indicator of a stable complex. **8c** had lower binding energy than acarbose; however, it was completely stable in the binding site of α-glucosidase. In fact, binding energy of − 75.1 kJ/mol was sufficient for making a stable complex between a small molecule like **8c** and α-glucosidase. Further inspection of free energy components revealed that molecular mechanics interaction energy (van der Waals energy + Electrostatic energy) was favorable and solvation energy (the sum of polar solvation energy and SASA energy) was unfavorable for the formation of both α-glucosidase–acarbose and α-glucosidase-**8c** complexes. In fact, Van der Waals and electrostatic energies were negative and solvation energy was positive in both complexes.

**Table 2:** Binding free energy (KJ/mol) for acarbose and **8c.**

Complex	Van der Waals energy	Electrostatic energy	Polar solvation energy	SASA^a^ energy	Binding energy
**8c**	− 166.2	− 27.3	138.0	− 19.6	− 75.1
Acarbose	− 219.9	− 30.1	163.8	− 29.5	− 115.7

^a^Solvent-accessible surface area.

### In vitro cytotoxicity

Cytotoxicity of the most potent new α-glucosidase inhibitors **8c**, **8e**, and **8g** was evaluated against a breast cancer cell line (MCF-7) and a normal human cell line (HDF) by MTT method^26^. The obtained data demonstrated that studied compounds did not show any cytotoxic activity against MCF-7 and HDF cell lines (IC_50_ > 200 μM) in comparison to the standard drug etoposide (IC_50_ = 12.4 ± 4.7 μM).

### In silico druglikeness, ADME, and Toxicity studies

In silico druglikeness/ADME/T prediction of the positive control acarbose and the most potent compound **8c** was performed by PreADMET online software and the obtained data were presented in Table 3.^27^ As can be seen in this table, acarbos did not follow of Lipinski 'Rule of five' while compound 8c followed of this rule. Acarbose and compound **8c** had poor permeability to Caco-2 cell. Permeability to blood brain barrier (BBB) and skin for acarbose and compound **8c** is in the acceptable range. Moreover, compound **8c** had high human intestinal absorption (HIA) while acarbose did not have HIA. In silico toxicity study also demonstrated that acarbose had carcinogenic effect on mouse and did not have this effect on rat while new compound **8c** did not have carcinogenic effect on mouse and rat.

**Table 3:** Druglikeness/ADME/T profile of acarbose and the most potent compound **8c**.

Druglikeness/ADME/T^a^	Compound	
	Acarbose	**8c**
Rule of Five	Violated	Suitable
Caco2	9.44448	1.37022
HIA	0.000000	87.699415
BBB	0.0271005	0.104802
Skin permeability	− 5.17615	− 4.62683
Carcino mouse	Positive	Negative
Carcino rat	Negative	Negative

^a^The recommended ranges for CaCO_2_: < 25 poor, > 500 great, HIA: > 80% is high < 25% is poor, BBB = − 3.0 to 1.2, and Skin_Permeability = − 8.0 to − 1.0.

## Conclusion

In this study, a new series of 5 benzo[d]imidazole-amide-1,2,3-triazole-*N*-arylacetamide hybrids **8a–n** was designed with consideration of the potent α-glucosidase inhibitor structures. These compounds were synthesized by simple chemical reactions and evaluated against α-glucosidase. Among these compounds, **8c**, **8e–h**, and **8k–m** showed significant activity in comparison to the positive control acarbose. The most potent compound **8c** was a comptituive inhibitor against α-glucosidase. According to in silico molecular studies, this compound with an acceptable energy interacted with important amino acids of the α-glucosidase active site. Compound **8c** was not cytotoxic in the in vitro and in silico studies. In silico studies also predicted that compound **8c** is orally active.

## Experimental

### General methods

All reactions were performed under aerobic atmosphere (in air). ^1^H NMR spectra were recorded using a Varian spectrometer 500 MHz instrument using DMSO-*d*_*6*_ as solvent with the Tetramethylsilane (TMS) as an internal standard. ^13^C NMR spectra were obtained at 125 MHz and referenced to the internal solvent signals. Chemical shifts were reported in parts per million (ppm) relative to TMS (*δ*). Multiplicities were indicated by s (singlet), d (doublet), t (triplet), q (quartet), m (multiplet), coupling constant *J* was reported in hertz (Hz). All the chemicals were purchased from Merck, Germany, and Sigma, Germany.

#### General procedure for the preparation of (1*H*-benzo[d]imidazol-2-yl)methanol 3

*O-*Phenylenediamine **1** (20 mmol) and glycolic acid **2** (60 mmol) were added to HCl (4 N, 10 ml) and refluxed for 24 h. After completion of the reaction, by dropwise addition of ammonia solution, a brown precipitate was formed that was filtrated and washed with water (20 ml) to give pure product **3**.

#### General procedure for the preparation of 1*H*-benzo[d]imidazole-2-carboxylic acid 4

Compound **3** (10 mmol) and NaOH (20 mmol in 20 ml water) were refluxed for 2 h. Next, KMnO_4_ (15 mmol) was added to the reaction mixture and refluxed for 16 h. After completion of the reaction, pH of mixture was adjusted on 5 and orange solid appeared, filtrated and dried to give pure acid **4**.

#### General procedure for the preparation of *N*-(prop-2-yn-1-yl)-1*H*-benzo[d]imidazole-2-carboxamide 6

For the synthesis of compound **6**, a mixture of 1*H*-benzo[d]imidazole-2-carboxylic acid **4** (3 mmol), TBTU (3.6 mmol), and DIEA (9 mmol) in DMF (4 ml) was stirred at room temperature for 20 min. Next, propargyl amine **5** (4.5 mmol) was added to the reaction mixture and the obtained mixture was stirred at room temperature for 24 h. After the completion of the reaction, indicated by TLC, the reaction mixture was quenched with water and a light brown precipitate **6** was filtrated and dried.

#### General procedure for the synthesis of benzo[d]imidazole-amide-1,2,3-triazole-*N*-arylacetamide hybrids 8a–n

A mixture of compounds **7a–n** (1.1 mmol**)**, sodium azide (0.9 mmol), and TEA (1.3 mmol) in DMF (4 ml) was stirred at room temperature for 30 min. Next, compound **6 (**1.1 mmol), CuSO_4_ (20 mol%), and sodium ascorbate (40 mol%) were added to the reaction mixture, and the obtained mixture was stirred at room temperature for 20–24 h. Upon completion of the reaction, examined by TLC, the reaction mixture was diluted with H_2_O (20 ml), poured in ice (20 g) and the final products **8a–n** were filtered of, washed with cold water, and extracted by EtOAc.

### *N*-((1-(2-oxo-2-(phenylamino)ethyl)-1*H*-1,2,3-triazol-4-yl)methyl)-1*H*-benzo[d]imidazole-2-carboxamide (8a)

White solid; Yield: 78%. m.p. 203–205 °C. IR (KBr, υ): 3346, 3283, 1663, 1324, 1169 cm^−1^. ^1^H NMR (500 MHz, DMSO-*d*_*6*_) δ 13.30 (s, 1H), 10.44 (s, 1H), 9.44 (t, *J* = 6.3 Hz, 1H), 8.03 (s, 1H), 7.73 (d, *J* = 8.0 Hz, 1H), 7.58 (d, *J* = 8.1 Hz, 2H), 7.55 (d, *J* = 7.2 Hz, 1H), 7.32 (m, 3H), 7.30–7.24 (t, *J* = 7.6 Hz, 1H), 7.08 (t, *J* = 7.5 Hz, 1H), 5.31 (s, 2H), 4.61 (d, *J* = 6.2 Hz, 2H). ^13^C NMR (125 MHz, DMSO-d_6_) δ 164.71, 159.27, 145.95, 145.04, 142.98, 138.89, 134.96, 129.37, 125.12, 124.61, 124.21, 123.03, 120.36, 119.67, 113.03, 52.64, 35.03. *Anal*. Calcd. for C_19_H_17_N_7_O_2_: C 60.79; H 4.56; N 26.12; Found: C 60.56; H 4.77; N 25.95.

### *N*-((1-(2-oxo-2-(*o*-tolylamino)ethyl)-1*H*-1,2,3-triazol-4-yl)methyl)-1*H*-benzo[d]imidazole-2-carboxamide (8b)

White solid; Yield: 76%. m.p. 206–208 °C. IR (KBr, υ): 3359, 3298, 1649, 1316, 1183 cm^−1^. ^1^H NMR (500 MHz, DMSO-*d*_*6*_) δ 13.30 (s, 1H), 9.75 (s, 1H), 9.43 (t, *J* = 6.2 Hz, 1H), 8.03 (s, 1H), 7.73 (d, *J* = 8.0 Hz, 1H), 7.55 (d, *J* = 7.9 Hz, 1H), 7.43 (d, *J* = 7.8 Hz, 1H), 7.32 (t, *J* = 7.5 Hz, 1H), 7.27 (t, *J* = 7.6 Hz, 1H), 7.22 (d, *J* = 7.4 Hz, 1H), 7.16 (t, *J* = 7.7 Hz, 1H), 7.09 (t, *J* = 7.5 Hz, 1H), 5.36 (s, 2H), 4.61 (d, *J* = 6.1 Hz, 2H), 2.22 (s, 3H). ^13^C NMR (125 MHz, DMSO-*d*_*6*_) δ 164.90, 159.27, 145.96, 145.04, 142.99, 135.99, 134.96, 132.03, 130.89, 126.52, 125.99, 125.19, 125.09, 124.61, 123.03, 120.37, 113.03, 52.36, 35.04, 18.25. *Anal*. Calcd. for C_20_H_19_N_7_O_2_: C 61.69; H 4.92; N 25.18; Found: C 61.41; H 5.15; N 25.38.

### *N*-((1-(2-oxo-2-(*m*-tolylamino)ethyl)-1*H*-1,2,3-triazol-4-yl)methyl)-1*H*-benzo[d]imidazole-2-carboxamide (8c)

White solid; Yield: 74%. m.p. 206–208 °C. IR (KBr, υ): 3352, 3293, 1653, 1348, 1193 cm^−1^. ^1^H NMR (500 MHz, DMSO-*d*_*6*_) δ 13.30 (s, 1H), 10.36 (s, 1H), 9.45 (d, *J* = 6.2 Hz, 1H), 8.03 (s, 1H), 7.73 (d, *J* = 7.9 Hz, 1H), 7.55 (d, *J* = 7.7 Hz, 1H), 7.42 (s, 1H), 7.34 (m, 2H), 7.28 (t, *J* = 7.8 Hz, 1H), 7.20 (t, *J* = 7.9 Hz, 1H), 6.89 (d, *J* = 7.5 Hz, 1H), 5.30 (s, 2H), 4.61 (d, *J* = 6.2 Hz, 2H), 2.26 (s, 3H). ^13^C NMR (125 MHz, DMSO-*d*_*6*_) δ 164.63, 159.27, 145.96, 145.04, 143.00, 138.81, 138.58, 134.96, 129.19, 125.10, 124.92, 124.60, 123.03, 120.37, 120.23, 116.87, 113.03, 52.66, 35.03, 21.61. *Anal*. Calcd. for C_20_H_19_N_7_O_2_: C 61.69; H 4.29; N 25.18; Found: C 61.46; H 4.57; N 25.03.

### *N*-((1-(2-oxo-2-(*p*-tolylamino)ethyl)-1*H*-1,2,3-triazol-4-yl)methyl)-1*H*-benzo[d]imidazole-2-carboxamide (8d)

White solid; Yield: 71%. m.p. 209–211 °C. IR (KBr, υ): 3363, 3301, 1652, 1320, 1196 cm^−1^. ^1^H NMR (500 MHz, DMSO-*d*_*6*_) δ 13.30 (s, 1H), 10.35 (s, 1H), 9.44 (t, *J* = 6.2 Hz, 1H), 8.03 (s, 1H), 7.73 (d, *J* = 8.0 Hz, 1H), 7.55 (d, *J* = 8.0 Hz, 1H), 7.46 (d, *J* = 8.2 Hz, 2H), 7.32 (t, *J* = 7.5 Hz, 1H), 7.27 (t, *J* = 7.6 Hz, 1H), 7.12 (d, *J* = 8.0 Hz, 2H), 5.29 (s, 2H), 4.61 (d, *J* = 6.1 Hz, 2H), 2.24 (s, 3H). ^13^C NMR (125 MHz, DMSO-*d*_*6*_) δ 164.44, 159.27, 145.96, 145.03, 142.99, 136.38, 134.96, 133.19, 129.73, 125.11, 124.60, 123.03, 120.37, 119.68, 113.03, 52.62, 35.03, 20.90. *Anal*. Calcd. for C_20_H_19_N_7_O_2_: C 61.69; H 4.92; N 25.18; Found: C 61.45; H 5.12; N 25.01.

### *N*-((1-(2-((2,3-dimethylphenyl)amino)-2-oxoethyl)-1*H*-1,2,3-triazol-4-yl)methyl)-1*H*-benzo[d]imidazole-2-carboxamide (8e)

White solid; Yield: 73%. m.p. 216–218 °C. IR (KBr, υ): 3357, 3292, 1646, 1314, 1165 cm^−1^. ^1^H NMR (500 MHz, DMSO-*d*_*6*_) δ 13.31 (s, 1H), 9.83 (s, 1H), 9.45 (t, *J* = 6.2 Hz, 1H), 8.05 (s, 1H), 7.74 (d, *J* = 8.0 Hz, 1H), 7.56 (d, *J* = 7.9 Hz, 1H), 7.32 (t, *J* = 7.5 Hz, 1H), 7.27 (t, *J* = 7.6 Hz, 1H), 7.18 (d, *J* = 7.6 Hz, 1H), 7.03 (d, *J* = 7.5, 1H), 7.01 (t, *J* = 7.5, 2H), 5.36 (s, 2H), 4.62 (d, *J* = 6.1 Hz, 2H), 2.23 (s, 3H), 2.09 (s, 3H). ^13^C NMR (125 MHz, DMSO-d_6_) δ 164.96, 159.29, 145.97, 145.06, 143.01, 137.59, 135.73, 134.97, 131.51, 127.73, 125.76, 125.10, 124.61, 123.73, 123.04, 120.38, 113.04, 52.36, 35.06, 20.56, 14.44. *Anal*. Calcd. for C_21_H_21_N_7_O_2_: C 62.52; H 5.25; N 24.30; Found: C 62.34; H 5.39; N 24.55.

### *N*-((1-(2-((2,4-dimethylphenyl)amino)-2-oxoethyl)-1*H*-1,2,3-triazol-4-yl)methyl)-1*H*-benzo[d]imidazole-2-carboxamide (8f)

White solid; Yield: 76%. m.p. 226–228 °C. IR (KBr, υ): 3360, 3294, 1649, 1335, 1208 cm^−1^. ^1^H NMR (500 MHz, DMSO-*d*_*6*_) δ 13.30 (s, 1H), 9.72 (s, 1H), 9.43 (d, *J* = 6.3 Hz, 1H), 8.03 (s, 1H), 7.73 (d, *J* = 8.0 Hz, 1H), 7.55 (d, *J* = 8.0 Hz, 1H), 7.32 (t, *J* = 7.5 Hz, 1H), 7.27 (t, *J* = 7.6 Hz, 1H), 7.06 (s, 3H), 5.35 (s, 2H), 4.60 (d, *J* = 6.1 Hz, 2H), 2.14 (s, 6H). ^13^C NMR (125 MHz, DMSO-*d*_*6*_) δ 164.53, 159.25, 145.94, 145.01, 143.98, 142.99, 135.54, 134.96, 134.67, 132.00, 128.19, 127.19, 125.05, 124.59, 123.02, 120.35, 113.02, 52.05, 35.03, 18.47. *Anal*. Calcd. for C_21_H_21_N_7_O_2_: C 62.52; H 5.25; N 24.30; Found: C 62.26; H 5.10; N 24.17.

### *N*-((1-(2-((2,6-dimethylphenyl)amino)-2-oxoethyl)-1*H*-1,2,3-triazol-4-yl)methyl)-1*H*-benzo[d]imidazole-2-carboxamide (8g)

White solid; Yield: 75%. m.p. 222–224 °C. IR (KBr, υ): 3355, 3280, 1642, 1332, 1190 cm^−1^. ^1^H NMR (500 MHz, DMSO-*d*_*6*_) δ 13.29 (s, 1H), 9.72 (s, 1H), 9.42 (t, *J* = 6.1 Hz, 1H), 8.02 (s, 1H), 7.72 (d, *J* = 8.1 Hz, 1H), 7.54 (d, *J* = 8.0 Hz, 1H), 7.32 (t, *J* = 7.5 Hz, 1H), 7.27 (t, *J* = 7.6 Hz, 1H), 7.06 (s, 3H), 5.34 (s, 2H), 4.60 (d, *J* = 6.1 Hz, 2H), 2.14 (s, 6H). ^13^C NMR (125 MHz, DMSO-*d*_*6*_) δ 164.53, 159.24, 145.92, 145.00, 142.96, 135.54, 134.67, 128.19, 127.18, 125.03, 124.59, 123.02, 120.35, 113.01, 52.04, 35.03, 18.47. *Anal*. Calcd. for C_21_H_21_N_7_O_2_: C 62.52; H 5.25; N 24.30; Found: C 62.28; H 5.47; N 24.11.

### *N*-((1-(2-((4-ethylphenyl)amino)-2-oxoethyl)-1*H*-1,2,3-triazol-4-yl)methyl)-1*H*-benzo[d]imidazole-2-carboxamide (8h)

White solid; Yield: 69%. m.p. 213–215 °C. IR (KBr, υ): 3344, 3281, 1648, 1357, 1279 cm^−1^. ^1^H NMR (500 MHz, DMSO-*d*_*6*_) δ 13. 27 (s, 1H), 10.34 (s, 1H), 9.41 (t, *J* = 6.1 Hz, 1H), 8.00 (s, 1H), 7.71 (d, *J* = 8.1 Hz, 1H), 7.53 (d, *J* = 8.0 Hz, 1H), 7.46 (d, *J* = 8.1 Hz, 2H), 7.31 (t, *J* = 7.5 Hz, 1H), 7.26 (t, *J* = 7.6 Hz, 1H), 7.14 (d, *J* = 8.1 Hz, 2H), 5.26 (s, 2H), 4.58 (d, *J* = 6.1 Hz, 2H), 2.53 (q, *J* = 7.7 Hz, 2H), 1.13 (t, *J* = 7.5 Hz, 3H). ^13^C NMR (125 MHz, DMSO-*d*_*6*_) δ 164.42, 159.23, 145.93, 145.00, 142.96, 139.61, 136.55, 134.93, 128.53, 125.08, 124.59, 123.02, 120.35, 119.74, 113.01, 52.59, 35.01, 28.03, 16.08. *Anal*. Calcd. for C_21_H_21_N_7_O_2_: C 62.52; H 5.25; N 24.30; Found: C 62.38; H 5.49; N 24.14.

### *N*-((1-(2-((4-methoxyphenyl)amino)-2-oxoethyl)-1*H*-1,2,3-triazol-4-yl)methyl)-1*H*-benzo[d]imidazole-2-carboxamide (8i)

White solid; Yield: 72%. m.p. 235–237 °C. IR (KBr, υ): 3356, 3287, 1650, 1331, 1189 cm^−1^. ^1^H NMR (500 MHz, DMSO-*d*_*6*_) *δ* 13.28 (s, 1H), 10.28 (s, 1H), 9.42 (t, *J* = 6.2 Hz, 1H), 8.01 (s, 1H), 7.71 (d, *J* = 8.0 Hz, 1H), 7.53 (d, *J* = 8.1 Hz, 1H), 7.47 (d, *J* = 8.5 Hz, 2H), 7.31 (t, *J* = 7.5 Hz, 1H), 7.26 (t, *J* = 7.7 Hz, 1H), 6.88 (d, *J* = 8.5 Hz, 2H), 5.25 (s, 2H), 4.59 (d, *J* = 6.0 Hz, 2H), 3.70 (s, 3H). ^13^C NMR (125 MHz, DMSO-*d*_*6*_) *δ* 164.17, 159.27, 156.00, 145.95, 145.02, 142.99, 134.95, 131.98, 125.09, 124.61, 123.04, 121.24, 120.37, 114.47, 113.03, 55.62, 52.57, 35.03. *Anal*. Calcd. for C_20_H_19_N_7_O_3_: C 59.25; H 4.72; N 24.18; Found: C 59.04; H 4.54; N 24.28.

### *N*-((1-(2-((4-fluorophenyl)amino)-2-oxoethyl)-1*H*-1,2,3-triazol-4-yl)methyl)-1*H*-benzo[d]imidazole-2-carboxamide (8j)

White solid; Yield: 75%. m.p. 245–247 °C. IR (KBr, υ): 3347, 3297, 1672, 1324, 1009 cm^−1^. ^1^H NMR (500 MHz, DMSO-*d*_*6*_) δ 13.27 (s, 1H), 10.48 (s, 1H), 9.42 (t, *J* = 6.2 Hz, 1H), 8.01 (s, 1H), 7.71 (d, *J* = 8.0 Hz, 1H), 7.57 (dd, *J* = 8.9, 4.9 Hz, 2H), 7.53 (d, *J* = 8.0 Hz, 1H), 7.31 (t, *J* = 7.5 Hz, 1H), 7.26 (t, *J* = 7.6 Hz, 1H), 7.15 (t, *J* = 8.7 Hz, 2H), 5.28 (s, 2H), 4.58 (d, *J* = 6.1 Hz, 2H). ^13^C NMR (125 MHz, DMSO-*d*_*6*_) δ 164.64, 161.56 (^1^*J*_C-F_ = 240 Hz), 159.24, 145.92, 145.04, 142.96, 135.25 (^4^*J*_C-F_ = 1.25 Hz), 134.92, 125.09, 124.60, 123.03, 121.52 (^3^*J*_C-F_ = 8.75 Hz), 121.45, 120.35, 116.05 (^2^*J*_C-F_ = 22.5 Hz), 113.02, 52.54, 35.01. *Anal*. Calcd. for C_19_H_16_FN_7_O_2_: C 58.01; H 4.10; N 24.92; Found: C 57.88; H 4.26; N 24.75.

### *N*-((1-(2-((4-chlorophenyl)amino)-2-oxoethyl)-1*H*-1,2,3-triazol-4-yl)methyl)-1*H*-benzo[d]imidazole-2-carboxamide (8k)

White solid; Yield: 71%. m.p. 236–238 °C. IR (KBr, υ): 3349, 3290, 1655, 1369, 767 cm^−1^. ^1^H NMR (500 MHz, DMSO-*d*_*6*_) δ 13.29 (s, 1H), 10.71 (s, 1H), 9.43 (t, *J* = 6.2 Hz, 1H), 8.02 (s, 1H), 7.72 (d, *J* = 8.0 Hz, 1H), 7.61 (d, *J* = 8.4 Hz, 2H), 7.54 (d, *J* = 8.0 Hz, 1H), 7.38 (d, *J* = 8.4 Hz, 2H), 7.34–7.25 (m, 2H), 5.32 (s, 2H), 4.59 (d, *J* = 6.1 Hz, 2H).^13^C NMR (125 MHz, DMSO-*d*_*6*_) δ 164.66, 160.03, 145.39, 145.04, 141.70, 137.88, 135.30, 129.26, 127.75, 125.10, 124.60, 123.01, 121.22, 120.10, 113.01, 52.61, 35.00. *Anal*. Calcd. for C_19_H_16_ClN_7_O_2_: C 55.68; H 3.94; N 23.92; Found: C 55.39; H 4.16; N 24.11.

### *N*-((1-(2-((2,4-dichlorophenyl)amino)-2-oxoethyl)-1*H*-1,2,3-triazol-4-yl)methyl)-1*H*-benzo[d]imidazole-2-carboxamide (8l)

White solid; Yield: 68%. m.p. 247–249 °C. IR (KBr, υ): 3337, 3288, 1674, 1306, 1178, 765 cm^−1^. ^1^H NMR (500 MHz, DMSO-d_6_) δ 13.28 (s, 1H), 10.10 (s, 1H), 9.43 (t, *J* = 6.2 Hz, 1H), 8.02 (s, 1H), 7.78 (d, *J* = 8.7 Hz, 1H), 7.72 (d, *J* = 7.9 Hz, 1H), 7.69 (s, 1H), 7.54 (d, *J* = 8.0 Hz, 1H), 7.41 (dd, *J* = 8.8, 2.4 Hz, 1H), 7.32 (t, *J* = 7.5 Hz, 1H), 7.27 (t*, J* = 7.6 Hz, 1H), 5.42 (s, 2H), 4.59 (d, *J* = 6.1 Hz, 2H). ^13^C NMR (125 MHz, DMSO-*d*_*6*_) δ 165.57, 159.24, 145.92, 145.08, 142.96, 134.92, 133.85, 130.21, 129.51, 128.14, 127.23, 125.13, 124.59, 123.02, 121.96, 120.35, 113.01, 52.33, 35.01. *Anal*. Calcd. for C_19_H_15_Cl_2_N_7_O_2_: C 51.37; H 3.40; N 22.07; Found: C 51.18; H 3.71; N 22.22.

### *N*-((1-(2-((4-bromophenyl)amino)-2-oxoethyl)-1*H*-1,2,3-triazol-4-yl)methyl)-1*H*-benzo[d]imidazole-2-carboxamide (8m)

White solid; Yield: 79%. m.p. 227–229 °C. IR (KBr, υ): 3353, 3285, 1649, 1342, 620 cm^−1^. ^1^H NMR (500 MHz, DMSO-*d*_*6*_) δ 13.29 (s, 1H), 10.58 (s, 1H), 9.43 (t, *J* = 6.3 Hz, 1H), 8.02 (s, 1H), 7.73 (d, *J* = 8.0 Hz, 1H), 7.65–7.38 (m, 5H), 7.30 (dt, *J* = 17.2, 7.4 Hz, 2H), 5.31 (s, 2H), 4.60 (d, *J* = 6.1 Hz, 2H). ^13^C NMR (125 MHz, DMSO-*d*_*6*_) δ 164.93, 159.24, 145.93, 145.06, 142.95, 138.24, 132.19, 125.10, 124.59, 123.03, 122.98, 121.61, 120.36, 115.83, 113.02, 52.62, 35.02. *Anal*. Calcd. for C_19_H_16_BrN_7_O_2_: C 50.23; H 3.55; N 21.58; Found: C 50.02; H 3.81; N 21.33.

### *N*-((1-(2-((4-nitrophenyl)amino)-2-oxoethyl)-1*H*-1,2,3-triazol-4-yl)methyl)-1*H*-benzo[d]imidazole-2-carboxamide (8n)

White solid; Yield: 71%. m.p. 259–261 °C. IR (KBr, υ): 3341, 3276, 1684, 1551, 1352, 1223 cm^−1^. ^1^H NMR (500 MHz, DMSO-*d*_*6*_) δ 13.29 (s, 1H), 11.05 (s, 1H), 9.41 (t, *J* = 6.3 Hz, 1H), 8.23 (d, *J* = 8.6 Hz, 2H), 8.05 (s, 1H), 7.82 (d, *J* = 8.7 Hz, 2H), 7.73 (d, *J* = 8.0 Hz, 1H), 7.54 (d, *J* = 8.0 Hz, 1H), 7.31 (d, *J* = 7.4 Hz, 1H), 7.28 (d, *J* = 7.5 Hz, 1H), 5.40 (s, 2H), 4.61 (d, *J* = 6.1 Hz, 2H). ^13^C NMR (125 MHz, DMSO-*d*_*6*_) δ 165.85, 159.25, 145.92, 145.14, 144.96, 143.03, 142.97, 134.87, 125.55, 125.15, 124.59, 123.01, 120.33, 119.48, 113.01, 52.73, 35.02. *Anal*. Calcd. for C_19_H_16_N_8_O_4_: C 54.29; H 3.84; N 26.66; Found: C 54.09; H 4.03; N 26.40.

### α-Glucosidase inhibition assay

In vitro anti-α-glucosidase inhibition and kinetic study of the new compounds **8a–n** were performed exactly according to our pervious reported works^23^.

### Docking study

Docking studies of the selected compounds **8b**, **8c**, and **8e** were performed on a homology model of α-glucosidase based on our pervious reported work^23^.

### Molecular dynamics

Molecular dynamics on the most potent compound **8c** and positive control acarbose were performed exactly according to our recently reported work^24^.

### Free Binding Energy calculations

Binding free energy calculation of protein–ligand complex was performed by using the g_mmpdsa program. This program was developed for calculation of components of binding free energy using the molecular mechanic/poisson-boltzmann surface area (MM/PBSA) method. This program calculates components of binding energy of protein–ligand complex which can be described as:

$$\begin{aligned} & {\text{Free binding energy}} = {\text{molecular mechanics interaction energy }}\left( {{\text{MMIE}}} \right) + {\text{solvation energy }}\left( {{\text{SE}}} \right) \\ & {\text{MMIE}} = {\text{van der Waals energy}} + {\text{Electrostatic energy}} \\ & {\text{SE}} = {\text{polar solvation energy }}\left( {{\text{PSE}}} \right) + {\text{nonpolar solvation energy }}\left( {\text{SASA energy}} \right) \\ & {\text{PSE}} = {\text{PSEcomplex}}{-}\left( {{\text{PSEprotein}} + {\text{PSEligand}}} \right) \\ & {\text{SASA energy}} = {\text{SASAcomplex}}{-}\left( {{\text{SASAprotein}} + {\text{SASAligand}}} \right) \\ \end{aligned}$$

Two hundred snapshots were taken at an interval of 100 ps during the last 20 ns period of MD trajectory and then binding energy calculations were performed.

### Evaluation of cytotoxic effects

Evaluation of in vitro cytotoxicity of the compounds **8c**, **8e**, and **8 g** was performed exactly based on a reported standard method^26^.

### In silico pharmacokinetic and toxicity predictions

In silico prediction of pharmacokinetic property and toxicity profile of acarbose and the most potent compound **8c** was performed using by the preADMET online server^27^.

## Supplementary Information

Supplementary Information.

## Data Availability

The datasets used or analyzed during the current study are available from the corresponding authors.
