# Supplementary material for: Design, synthesis, in vitro, and in silico evaluations of benzo[d]imidazole-amide-1,2,3-triazole-N-arylacetamide hybrids as new antidiabetic agents targeting α-glucosidase
Source: Sci Rep. 2023 Jul 31;13:12397. doi: 10.1038/s41598-023-39424-8 (PMC10390517; doi:10.1038/s41598-023-39424-8)
Supplement: Supplementary file 1 — Supplementary Information. [file 41598_2023_39424_MOESM1_ESM.docx]

**Design, synthesis, *in vitro*, and *in silico* evaluations of benzo[d]imidazole-amide-1,2,3-triazole-*N*-arylacetamide hybrids as new antidiabetic agents targeting α-glucosidase**

Faeze Yousefnejad^1^, Mahyar Mohammadi-Moghadam-Goozali^2^, Mohammad Hossein Sayahi^3^, Mohammad Halimi^4^, Ali Moazzam^1^, Maryam Mohammadi-Khanaposhtani^5^, Somayeh Mojtabavi^6^, Mehdi Asadi^7^, Mohammad Ali Faramarzi^6^, Bagher Larijani^1^, Massoud Amanlou^2, ^[[1]](#footnote-1)^*^, Mohammad Mahdavi^1, ^[[2]](#footnote-2)^*^

^1^ Endocrinology and Metabolism Research Center, Endocrinology and Metabolism Clinical Sciences Institute, Tehran University of Medical Sciences, Tehran, Iran ^✉^e-mail: [momahdavi@tums.ac.ir](mailto:momahdavi@tums.ac.ir) (M. Mahdavi).

^2^ Department of Medicinal Chemistry, Faculty of Pharmacy, Tehran University of Medical Sciences, Tehran, Iran ^✉^e-mail: [amanlou@tums.ac.ir](mailto:amanlou@tums.ac.ir) (M. Amanlou)

^3^ Department of Chemistry, Payame Noor University (PNU), P.O. Box 19395-3697, Tehran, Iran

^4^ Department of Biology, Babol Branch, Islamic Azad University, Babol, Iran

^5^ Cellular and Molecular Biology Research Center, Health Research Institute, Babol University of Medical Sciences, Babol, Iran

^6^ Department of Pharmaceutical Biotechnology, Faculty of Pharmacy, Tehran University of Medical Sciences, Tehran, Iran

^7^ Department of Medicinal Chemistry, Faculty of Pharmacy, Iran University of Medical Sciences, Tehran, Iran

**Supporting Information**

*N*-((1-(2-oxo-2-(phenylamino)ethyl)-1*H*-1,2,3-triazol-4-yl)methyl)-1*H*-benzo[d]imidazole-2-carboxamide **(8a)**


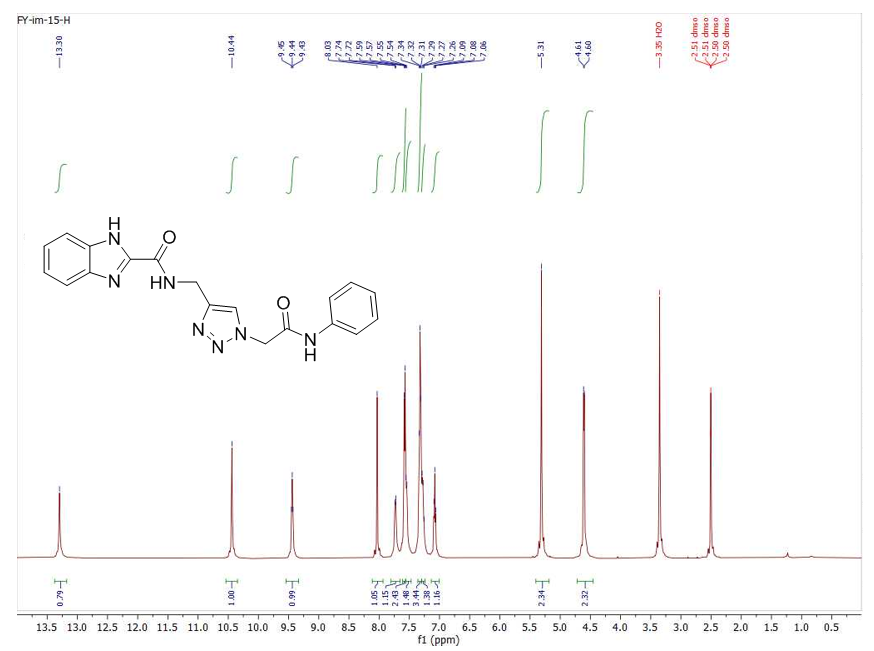


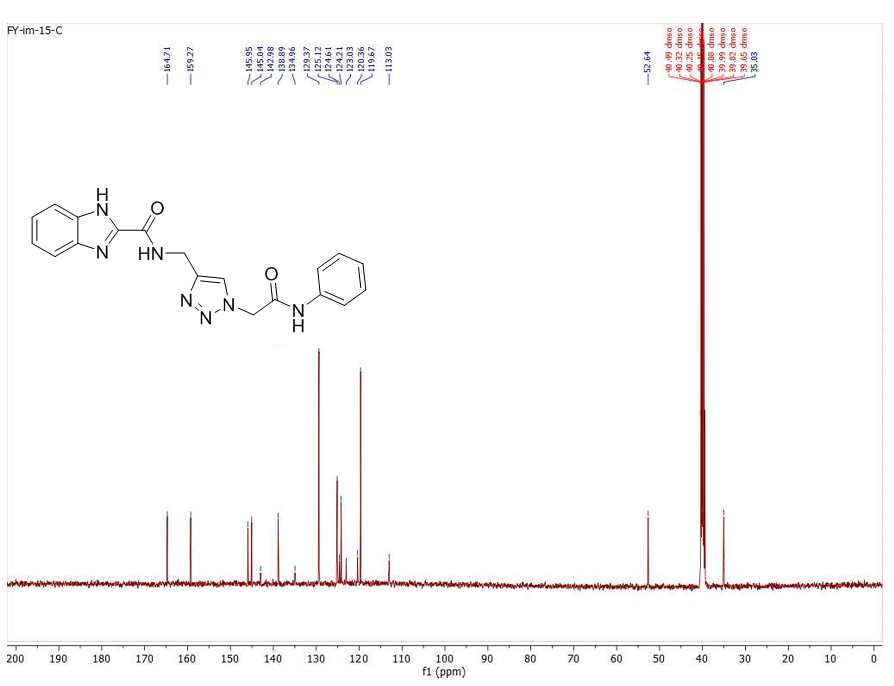


*N-((1-(2-oxo-2-(o-tolylamino)ethyl)-1H-1,2,3-triazol-4-yl)methyl)-1H-benzo[d]imidazole-2-carboxamide (****8b****)*


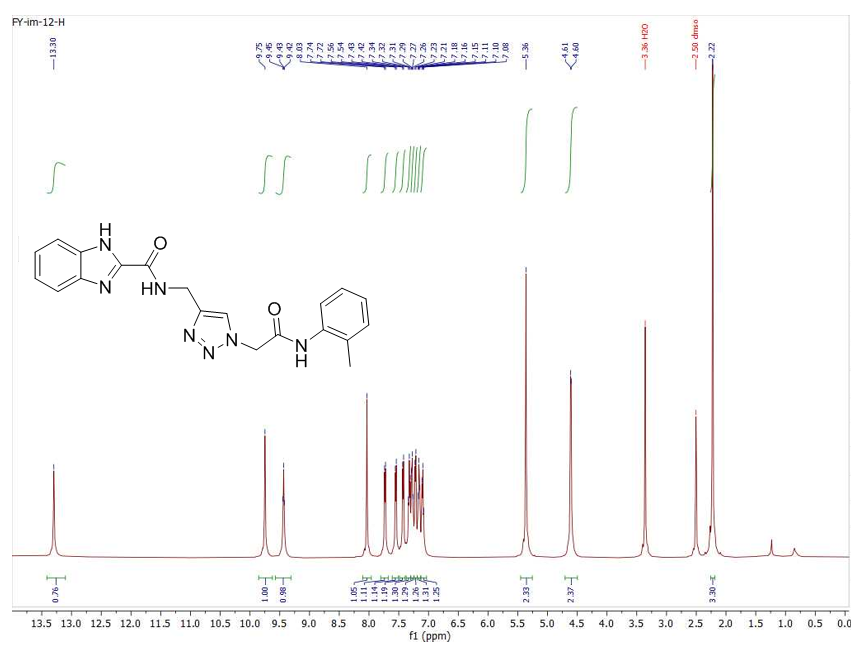


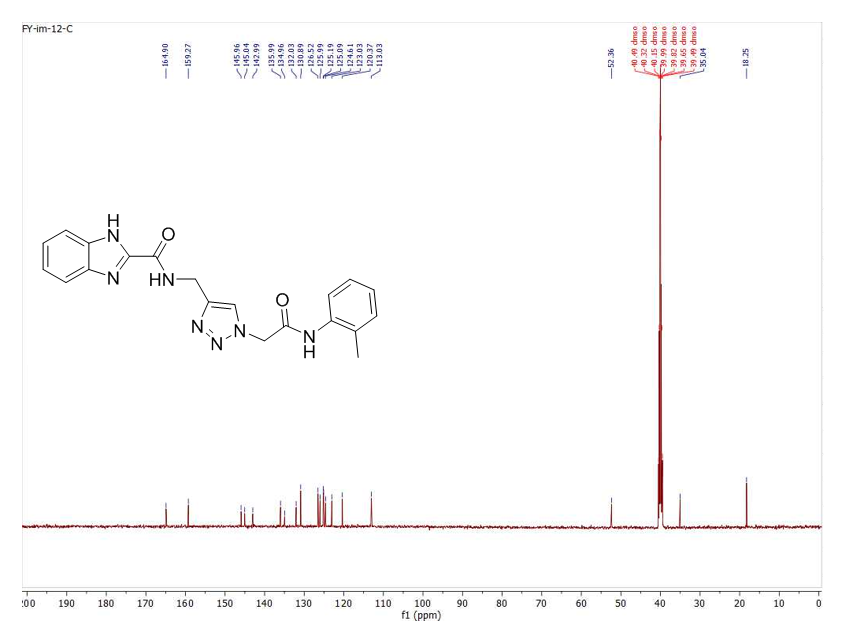


*N*-((1-(2-oxo-2-(m-tolylamino)ethyl)-1*H*-1,2,3-triazol-4-yl)methyl)-1*H*-benzo[d]imidazole-2-carboxamide **(8c)**


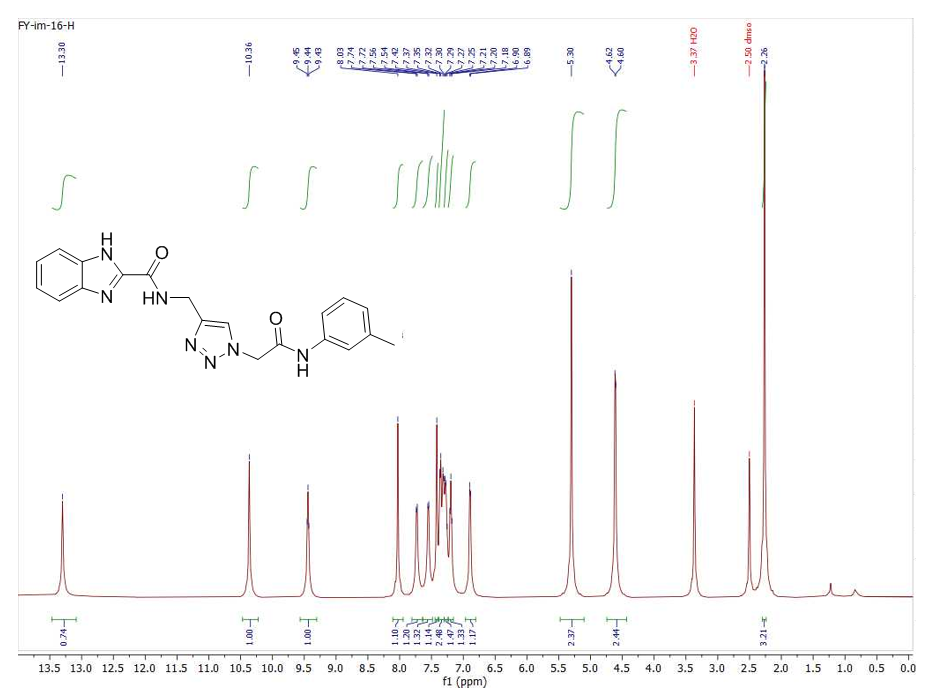


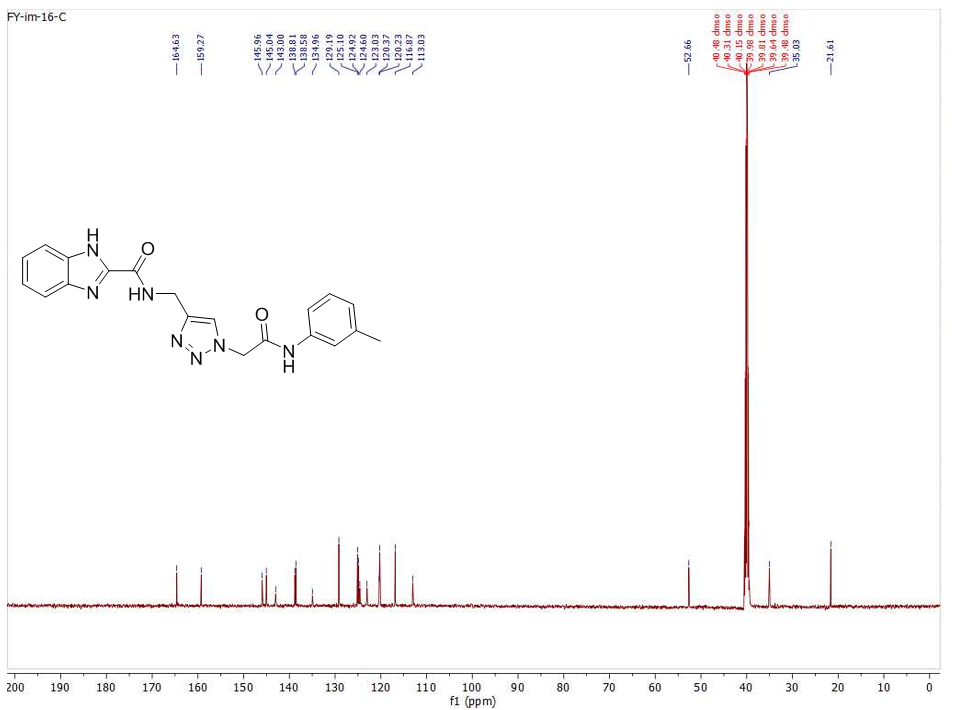


*N-((1-(2-oxo-2-(p-tolylamino)ethyl)-1H-1,2,3-triazol-4-yl)methyl)-1H-benzo[d]imidazole-2-carboxamide (****8d****)*


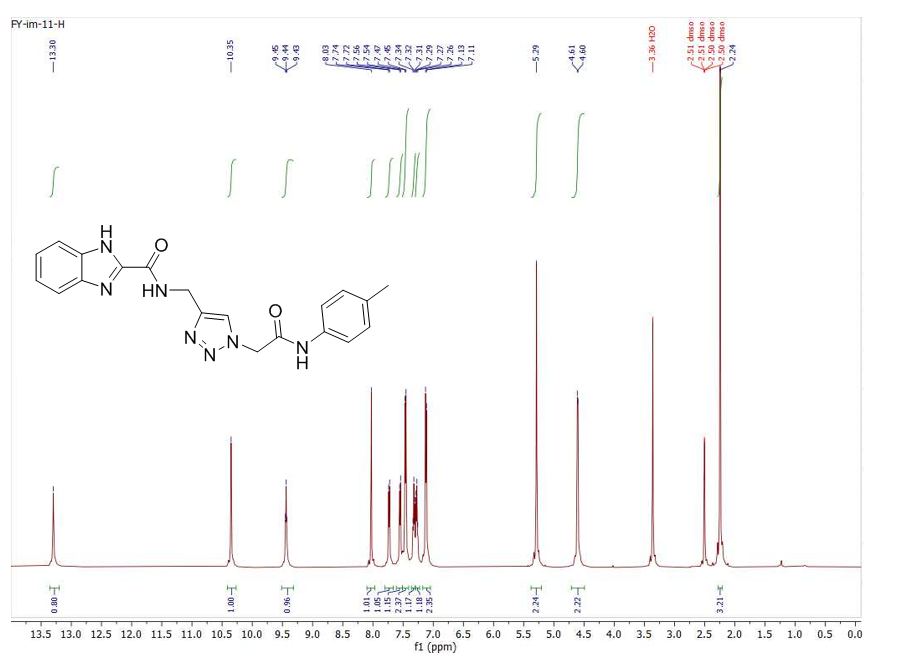


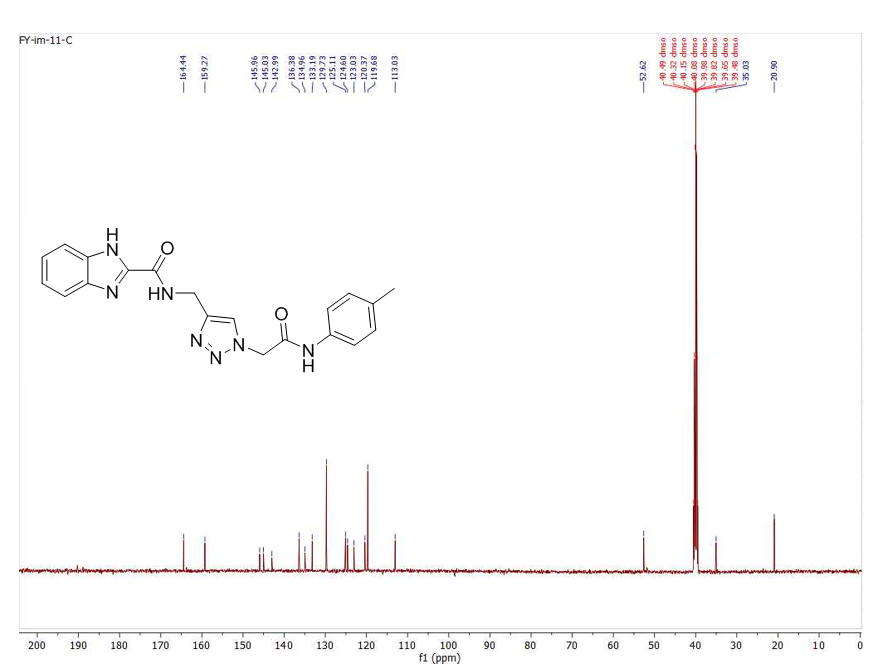


*N-((1-(2-((2,3-dimethylphenyl)amino)-2-oxoethyl)-1H-1,2,3-triazol-4-yl)methyl)-1H-benzo[d]imidazole-2-carboxamide (****8e****)*


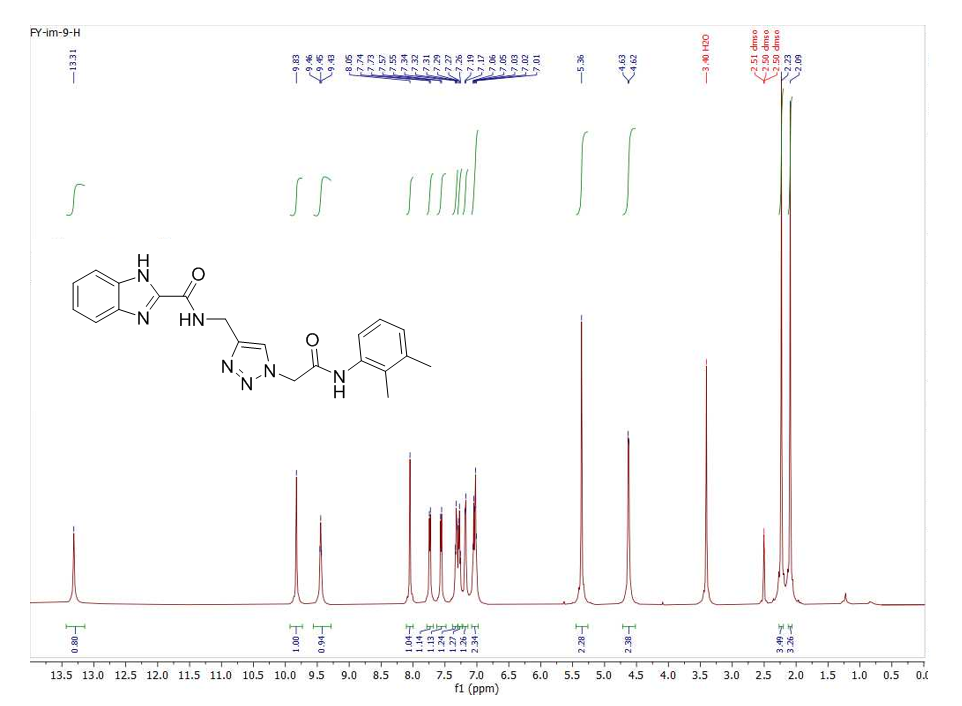


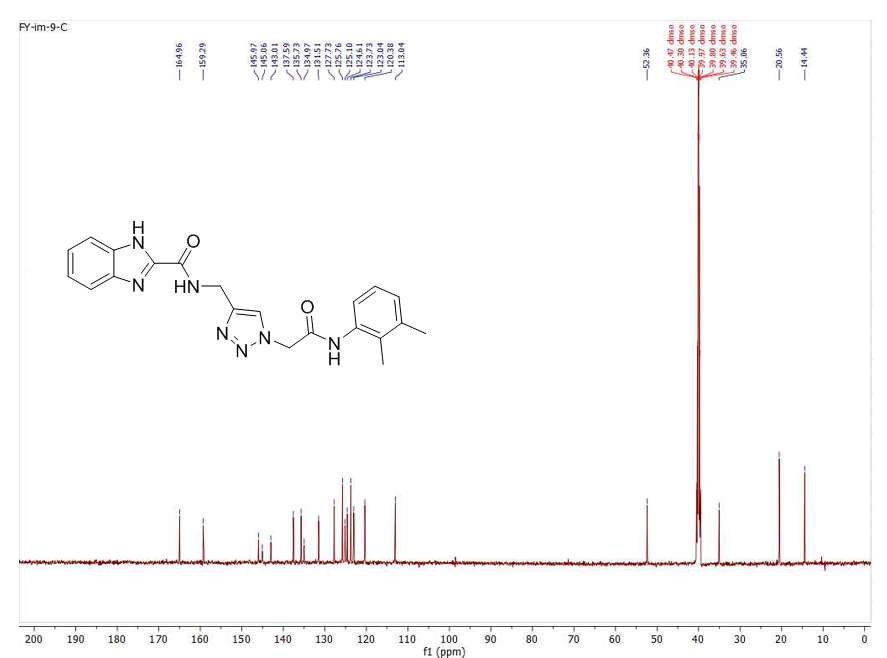


*N-((1-(2-((2,4-dimethylphenyl)amino)-2-oxoethyl)-1H-1,2,3-triazol-4-yl)methyl)-1H-benzo[d]imidazole-2-carboxamide (****8f****)*


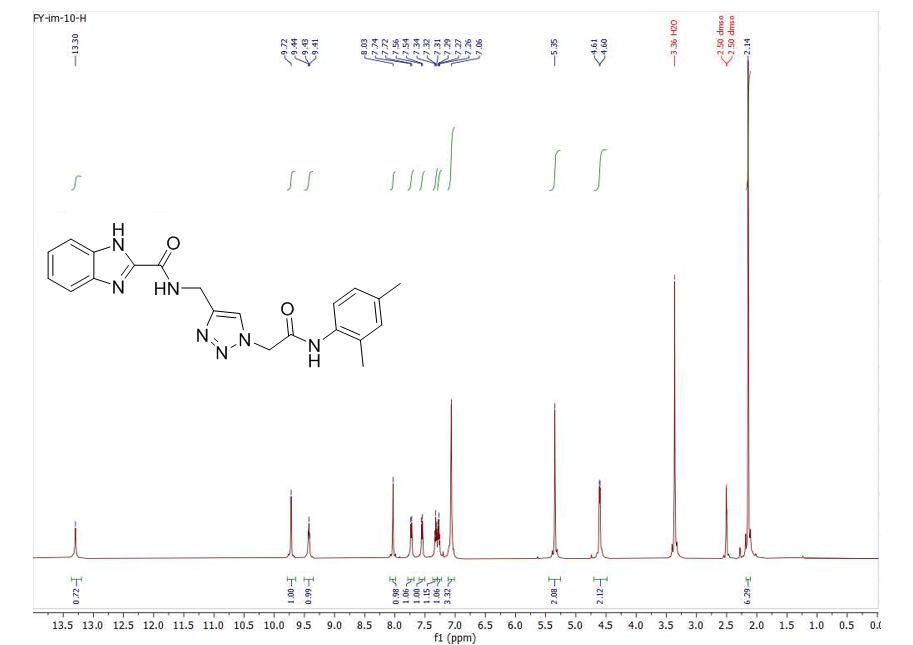


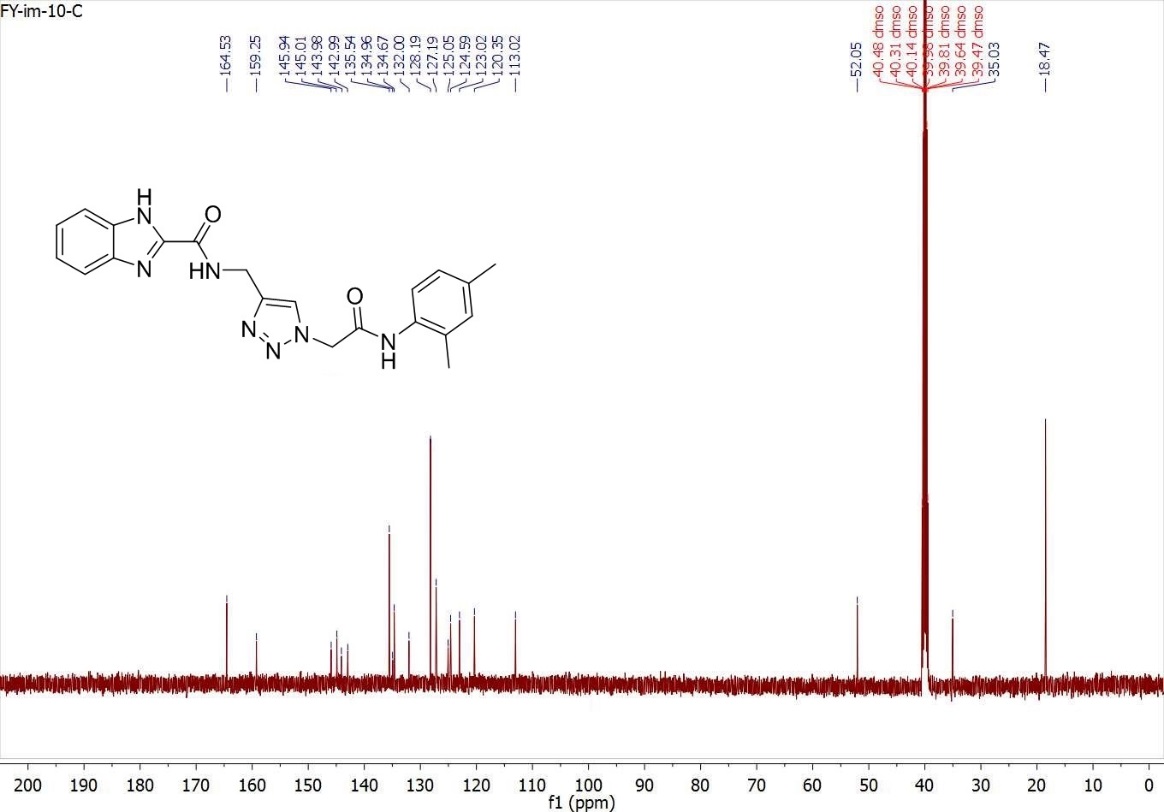


*N-((1-(2-((2,6-dimethylphenyl)amino)-2-oxoethyl)-1H-1,2,3-triazol-4-yl)methyl)-1H-benzo[d]imidazole-2-carboxamide (****8g****)*


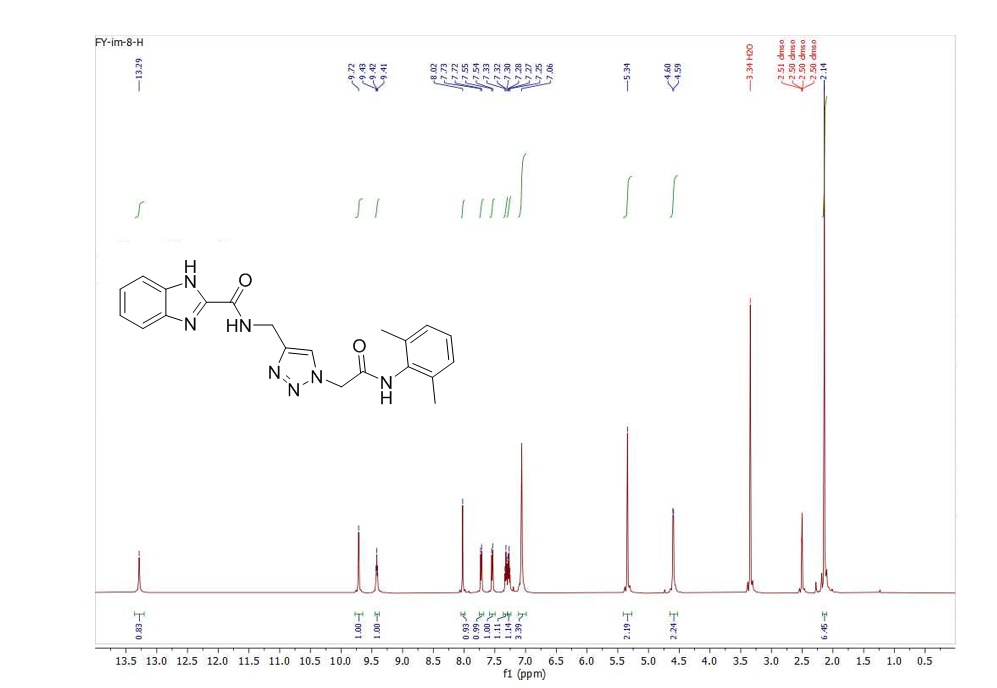


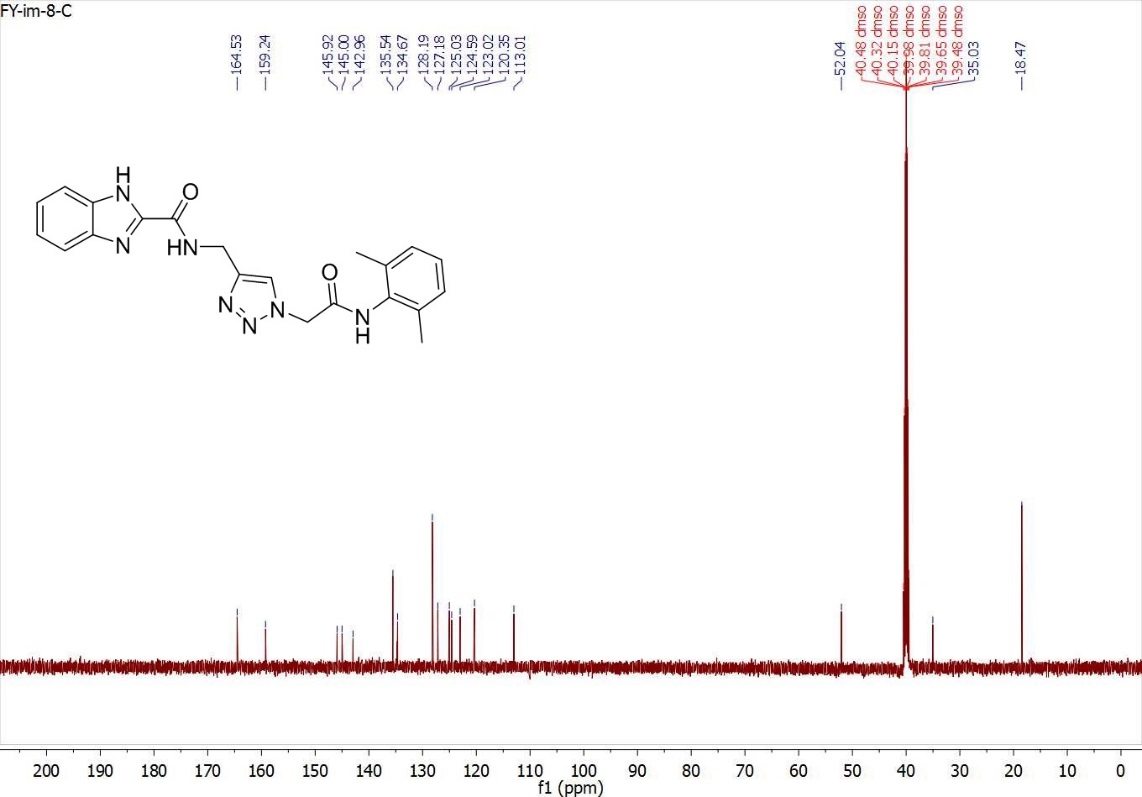


*N-((1-(2-((4-ethylphenyl)amino)-2-oxoethyl)-1H-1,2,3-triazol-4-yl)methyl)-1H-benzo[d]imidazole-2-carboxamide (****8h****)*


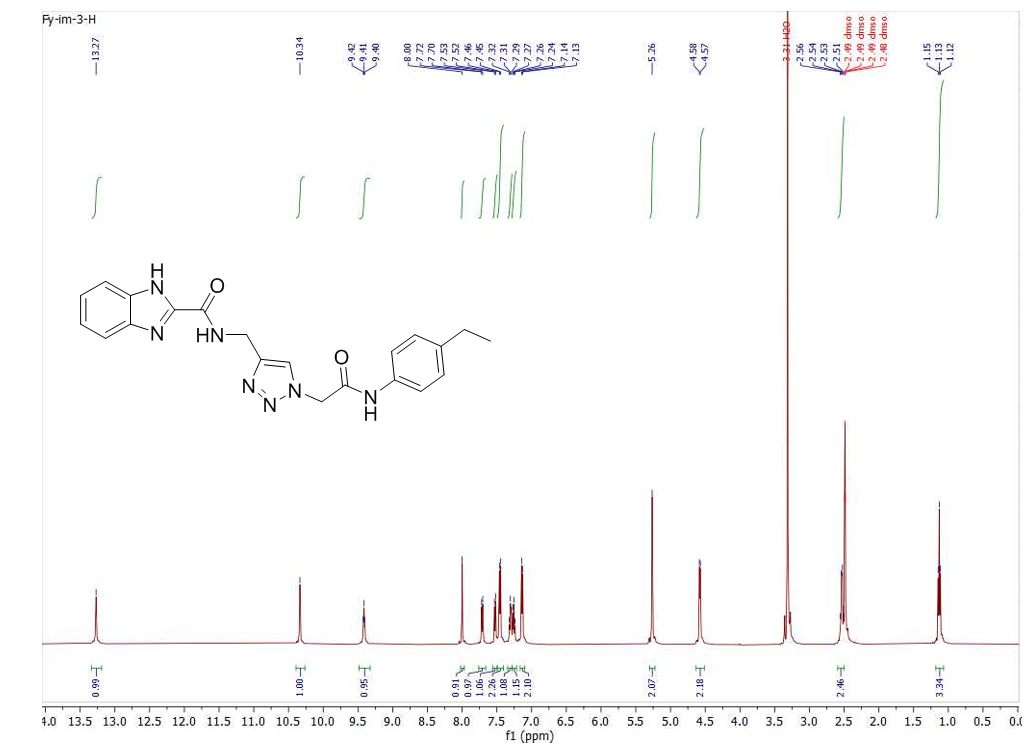


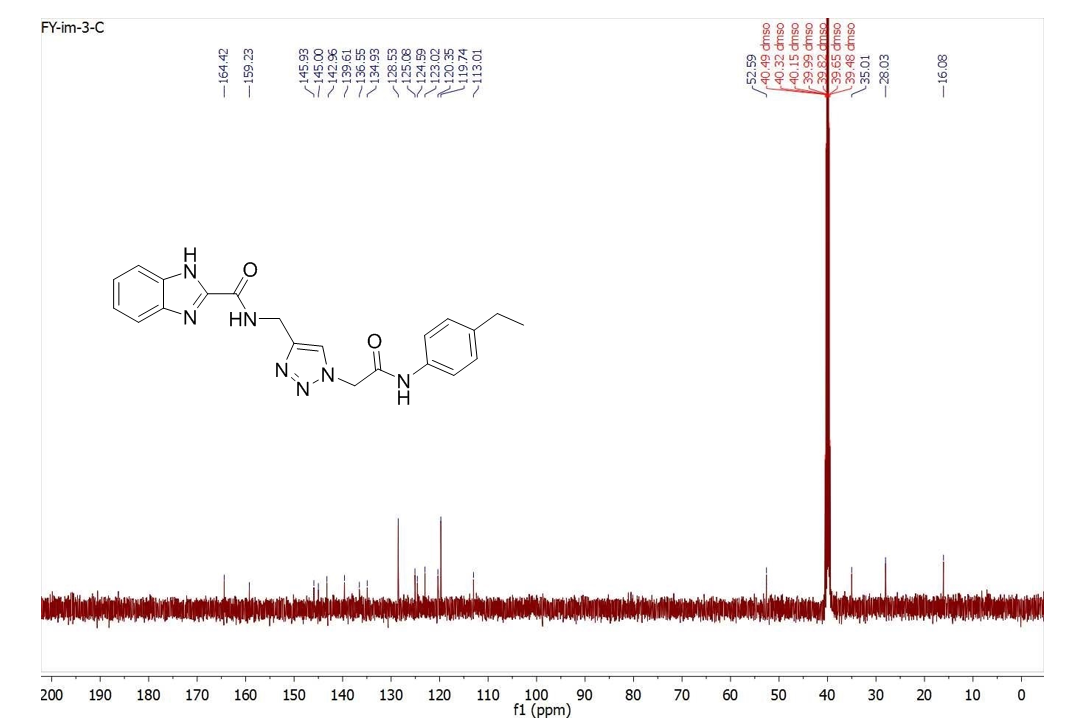


*N-((1-(2-((4-methoxyphenyl)amino)-2-oxoethyl)-1H-1,2,3-triazol-4-yl)methyl)-1H-benzo[d]imidazole-2-carboxamide (****8i****)*


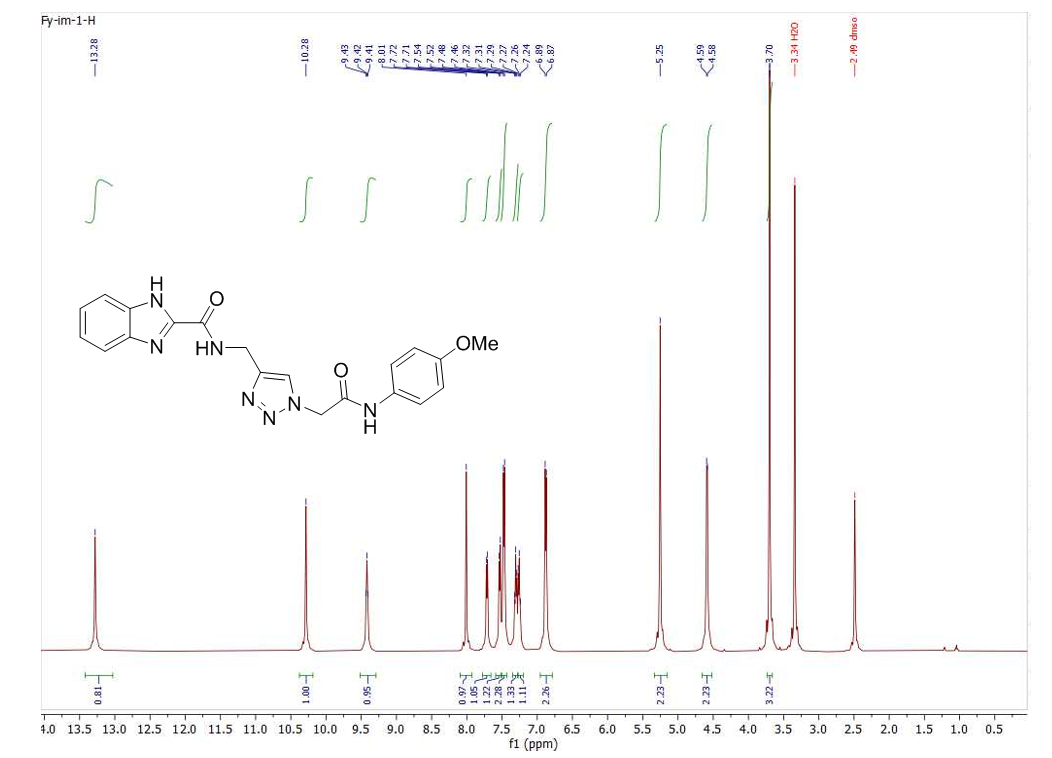


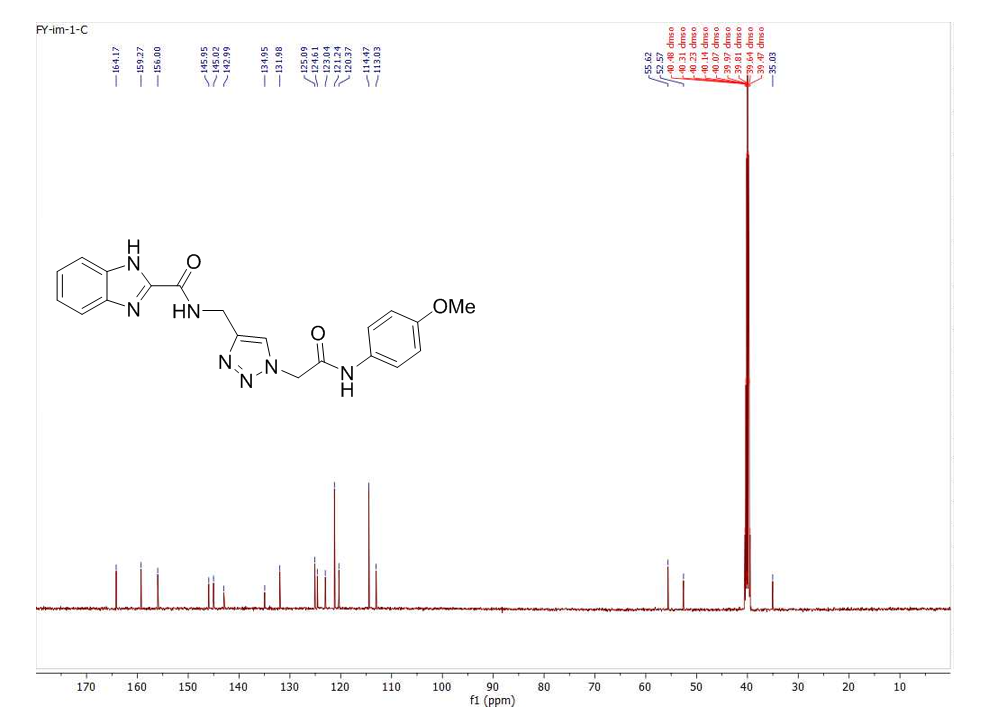


*N-((1-(2-((4-fluorophenyl)amino)-2-oxoethyl)-1H-1,2,3-triazol-4-yl)methyl)-1H-benzo[d]imidazole-2-carboxamide (****8j****)*


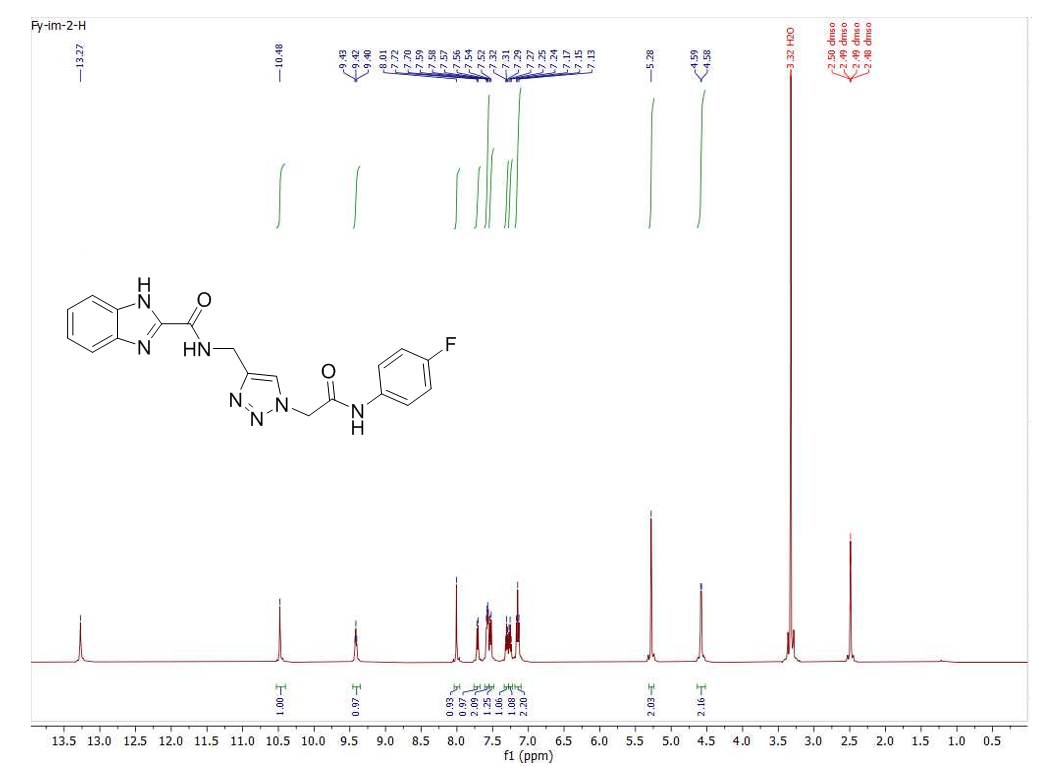


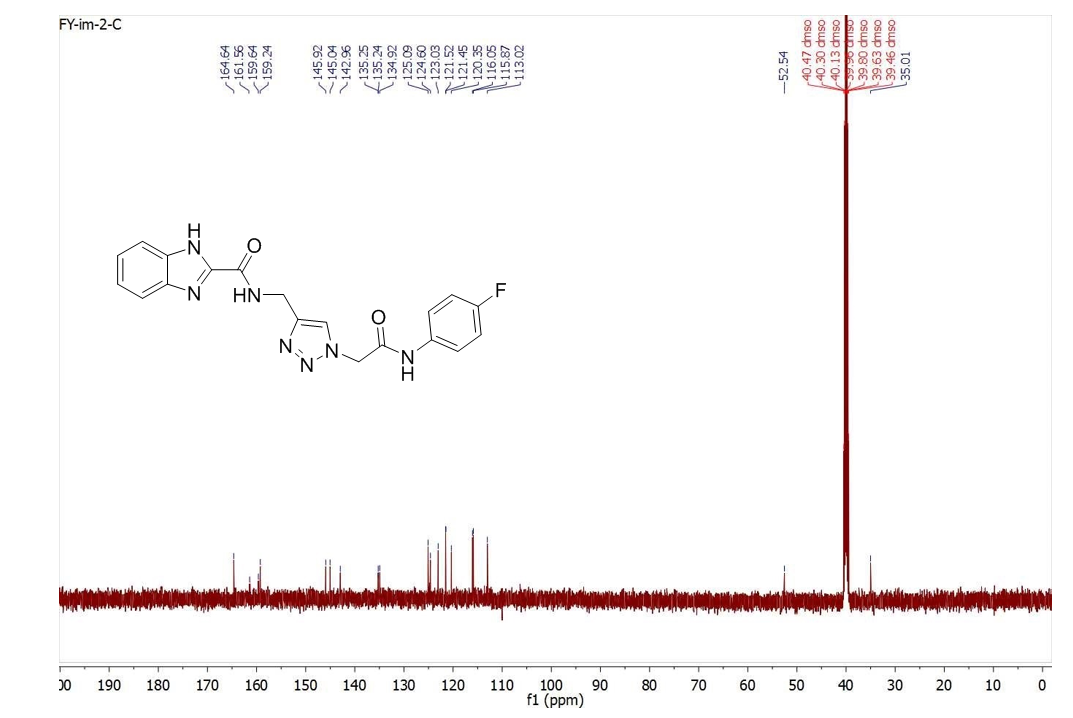


*N-((1-(2-((4-chlorophenyl)amino)-2-oxoethyl)-1H-1,2,3-triazol-4-yl)methyl)-1H-benzo[d]imidazole-2-carboxamide (****8k****)*


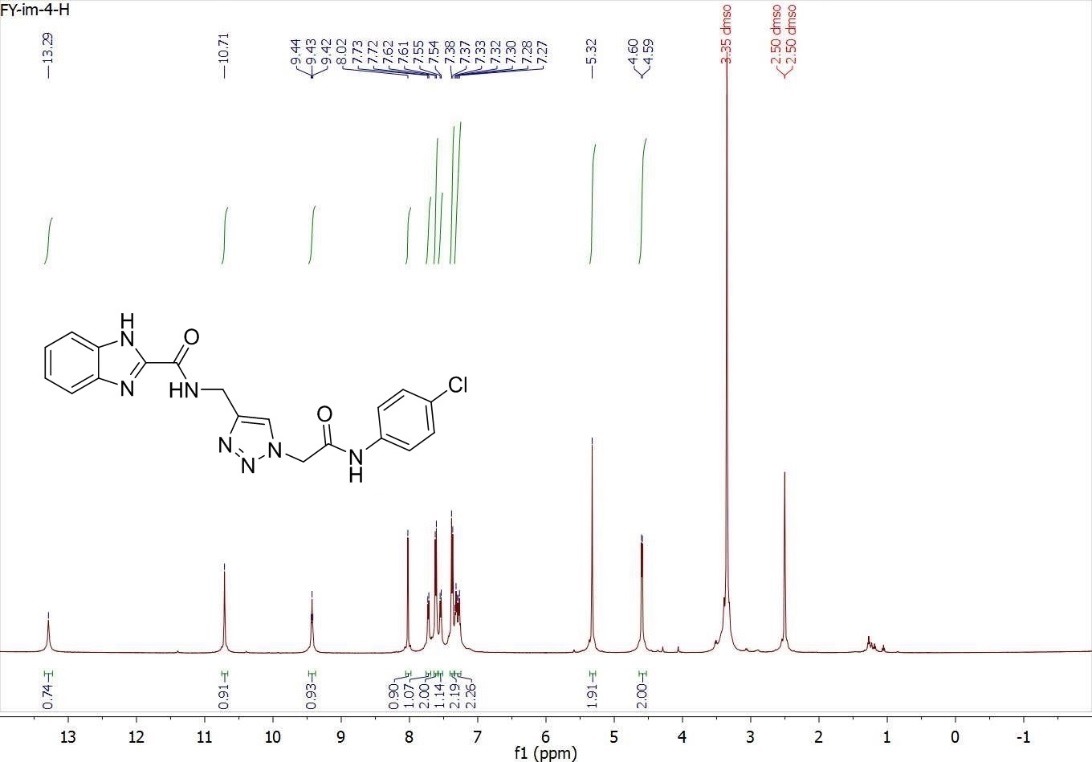


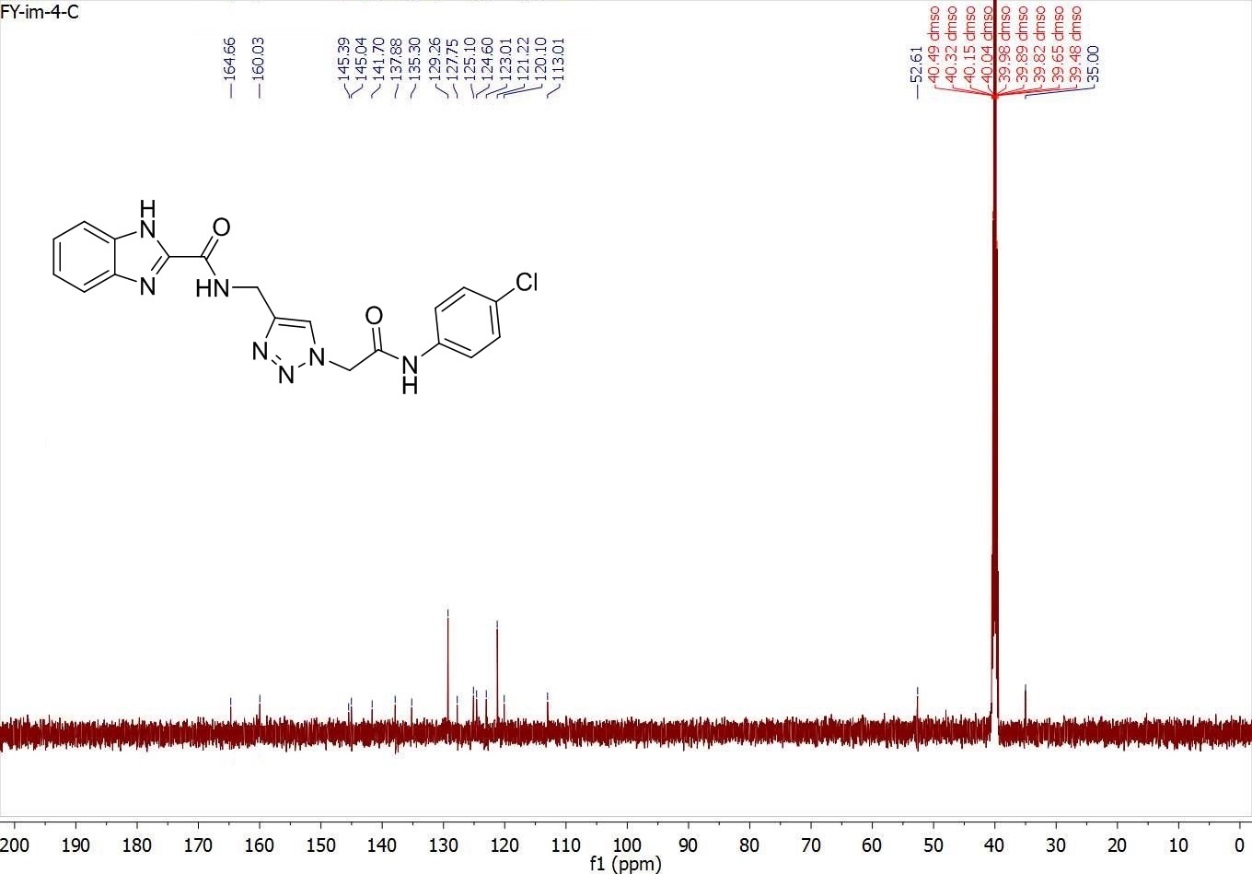


*N*-((1-(2-((2,4-dichlorophenyl)amino)-2-oxoethyl)-1H-1,2,3-triazol-4-yl)methyl)-1H-benzo[d]imidazole-2-carboxamide **(8l)**


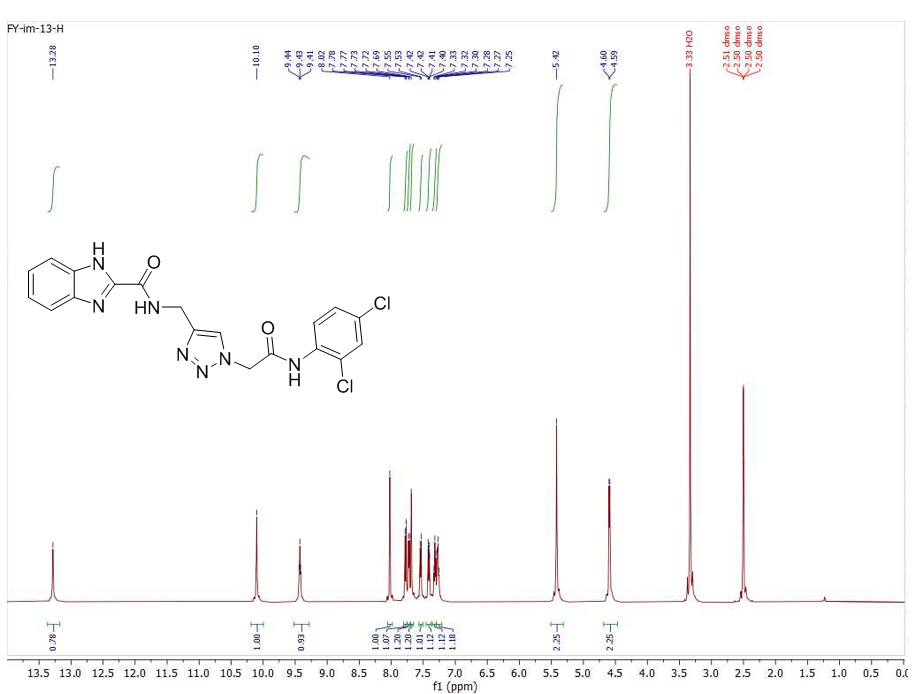


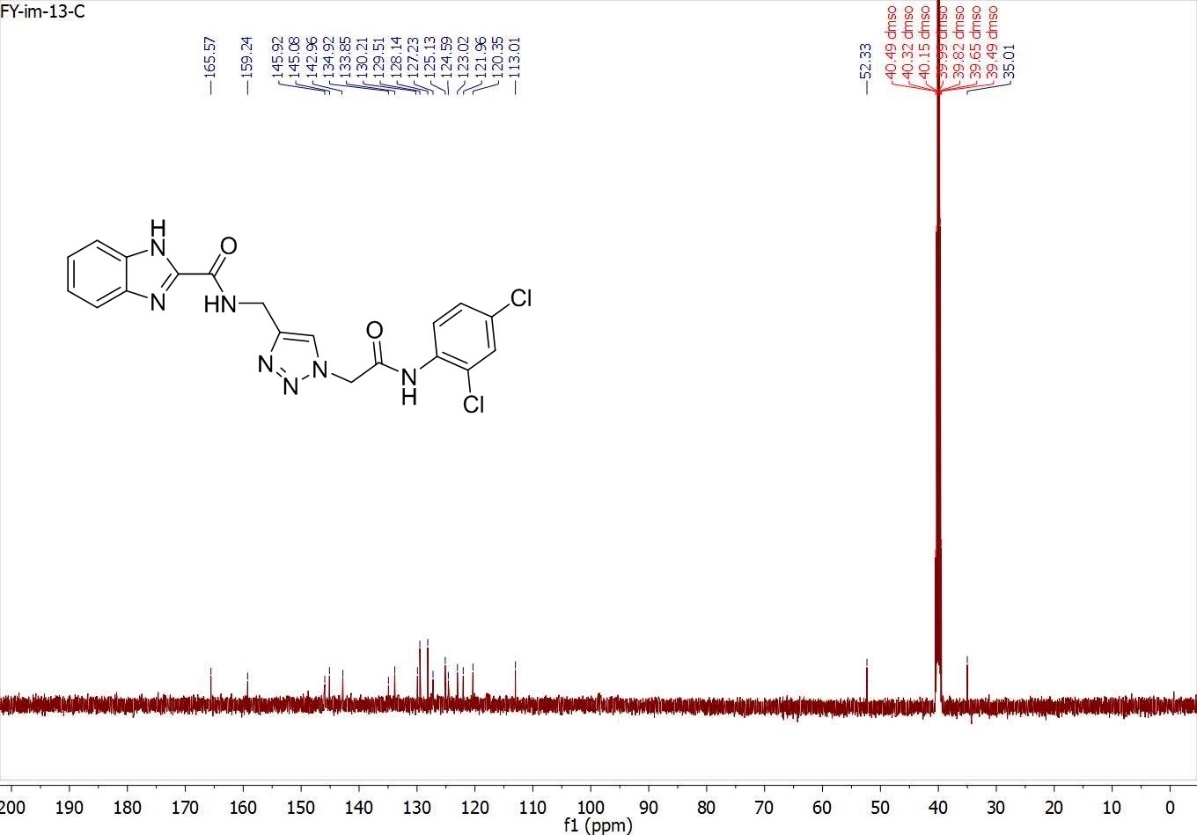


*N-((1-(2-((4-bromophenyl)amino)-2-oxoethyl)-1H-1,2,3-triazol-4-yl)methyl)-1H-benzo[d]imidazole-2-carboxamide (****8m****)*


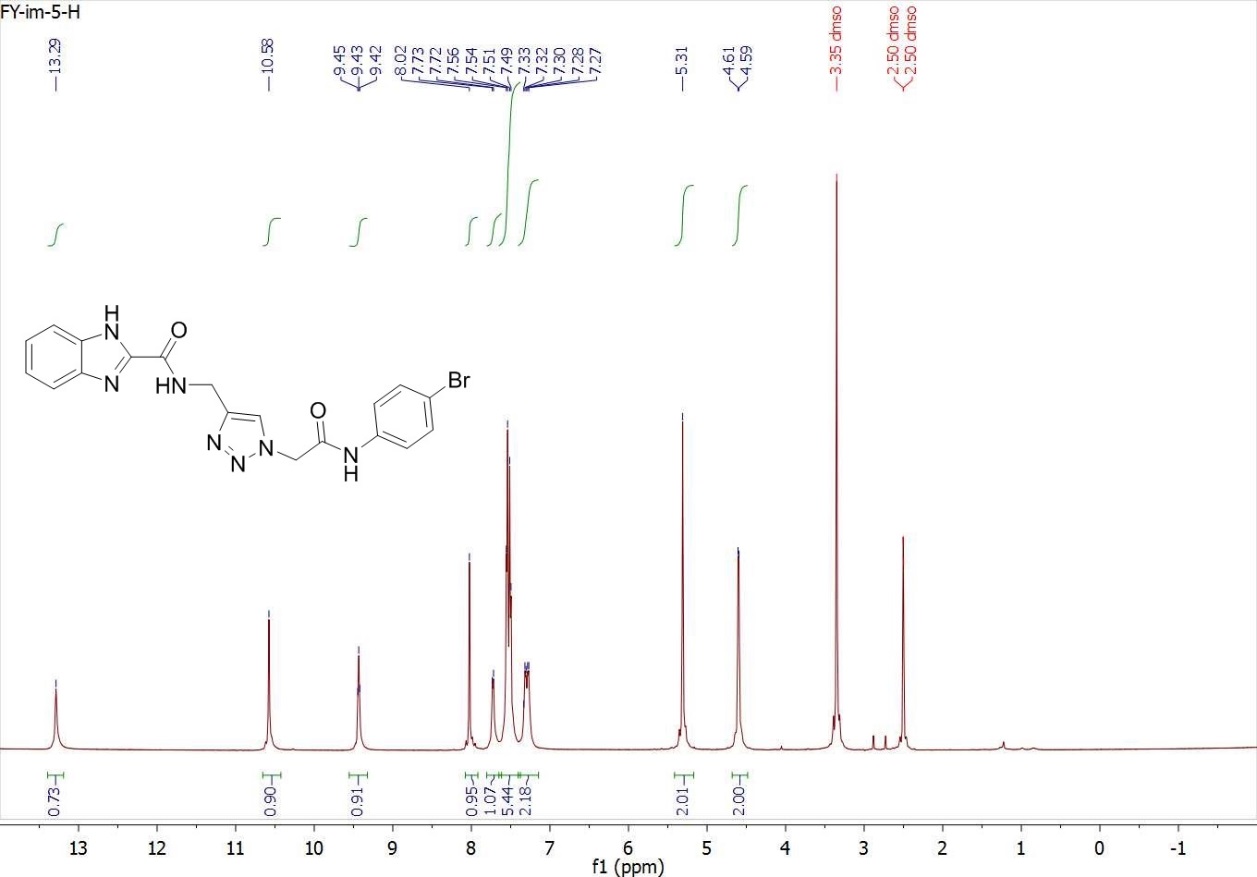


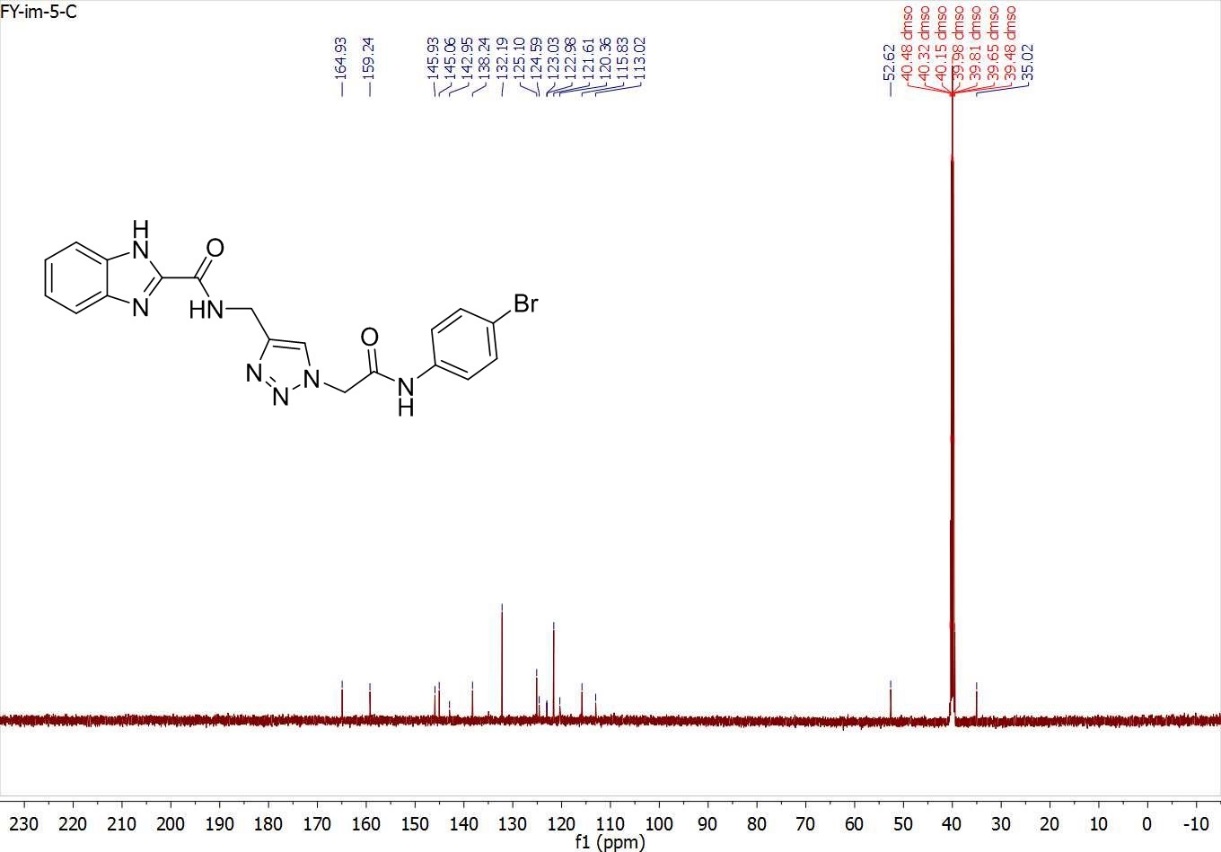


*N-((1-(2-((4-nitrophenyl)amino)-2-oxoethyl)-1H-1,2,3-triazol-4-yl)methyl)-1H-benzo[d]imidazole-2-carboxamide (****8n****)*

***
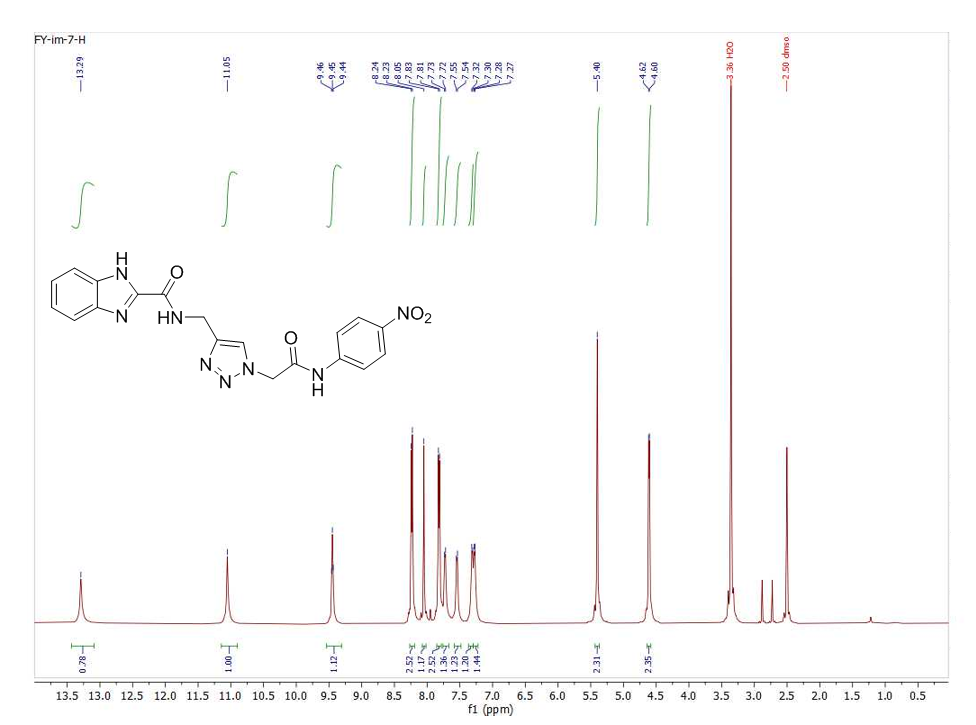
***


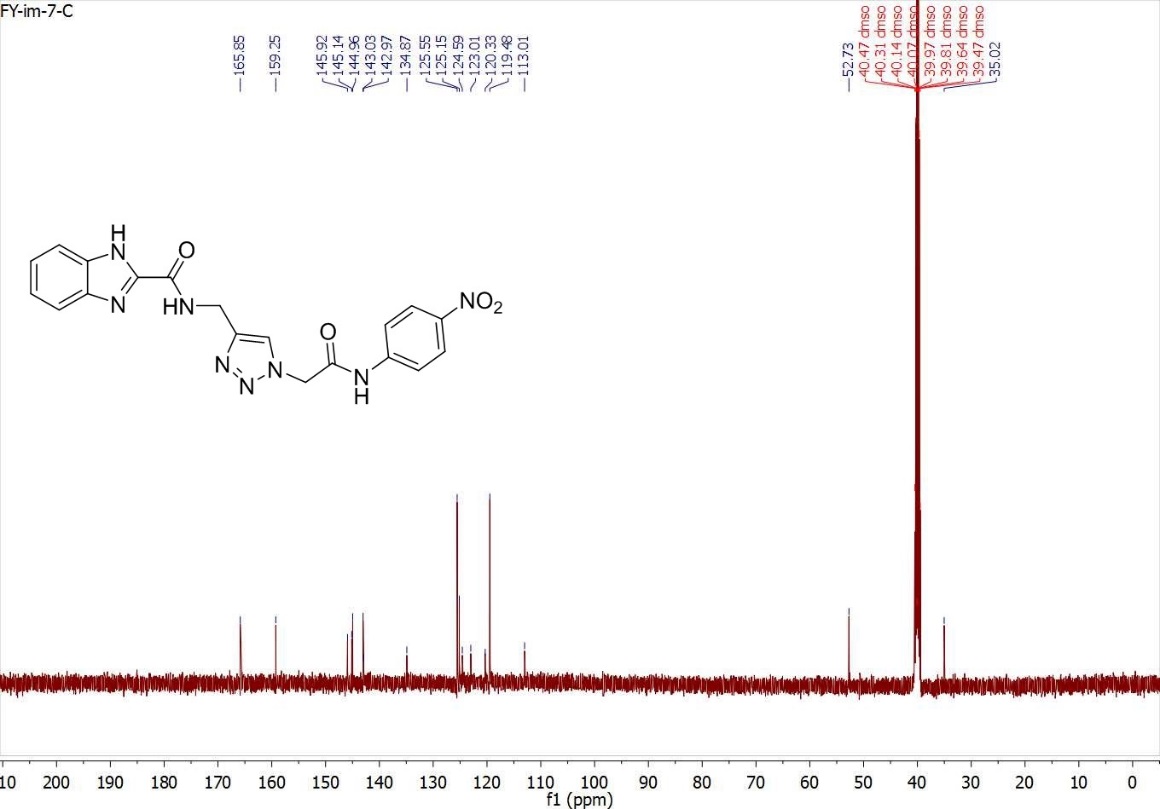


1. * Correspondences: Massoud Amanlou, E-mail: amanlou@tums.ac.ir, Tel.: +982166959067; Fax: +982164121111 and Mohammad Mahdavi, E-mail: momahdavi@sina.tums.ac.ir, Tel.: +982166954708; Fax: +982166461178. [↑](#footnote-ref-1)
2. [↑](#footnote-ref-2)
